# Dietary Stevia-Derived Isochlorogenic Acid Attenuates LPS-Induced Ileal Injury and Is Associated with Altered TLR4/MyD88/NF-κB and Nrf2/HO-1 Gene Expression in Weaned Lambs

**DOI:** 10.3390/ani16203256

**Published:** 2026-10-16

**Authors:** Ziyi Liang, Yuting Yuan, Qinghong Hao, Linzhi Xia, Xiaomin An, Ziwei Li, Kai Zhang, Yueqin Liu, Yingjie Zhang, Yunxia Guo

**Affiliations:** 1 College of Life Sciences, Hebei Agricultural University, Baoding 071001, China; 15931064933@163.com (Z.L.); yuanyyt7323@163.com (Y.Y.); hqh1128@163.com (Q.H.); m13934687952@163.com (L.X.); aaxm888@163.com (X.A.); 15533912716@163.com (Z.L.); 13171872170@163.com (K.Z.); 2 College of Animal Science and Technology, Hebei Agricultural University, Baoding 071001, China; liuyueqin66@126.com (Y.L.); zhangyingjie66@126.com (Y.Z.)

**Keywords:** weaned lambs, ICGA, oxidative stress, intestinal health, TLR4/MyD88/NF-κB signaling pathway

## Abstract

Weaning stress-induced oxidative damage impairs lamb growth and health. This study explored the protective effects of stevia-derived isochlorogenic acid (ICGA) against lipopolysaccharide (LPS)-induced injury in 48 weaned lambs (50 ± 2 d old, 15 ± 2.30 kg), randomly allocated to four groups. CON and LPS groups received a basal diet; ICGA1 and ICGA2 received a basal diet with 1.5 and 2.0 g/kg ICGA for 14 d. On day 15, LPS (200 μg/kg BW) or saline was injected intraperitoneally. ICGA showed a trend to increase ADG (*p* > 0.05). ICGA reduced serum IL-6, TNF-α, IL-1β, DAO, D-LA, ET, and COR; elevated immunoglobulins; improved ileal morphology; upregulated tight junction genes and Nrf2/HO-1 pathway genes; and suppressed the TLR4/MyD88/NF-κB pathway. ICGA also modulated gut microbiota. In conclusion, ICGA alleviates inflammation and oxidative stress and improves intestinal barrier function in weaned lambs.

## Simple Summary

Weaning stress triggers oxidative damage and impairs growth performance and health in lambs. Isochlorogenic acid possesses anti-inflammatory and antioxidant bioactivities, making it a promising functional feed additive to modulate animal immunity. This study aimed to assess the protective effects of stevia-derived isochlorogenic acid against lipopolysaccharide-induced oxidative injury and to uncover its underlying molecular mechanisms. Forty-eight weaned lambs were assigned to four groups; the two Isochlorogenic acid groups received graded dietary Isochlorogenic acid supplementation before acute lipopolysaccharide inflammatory challenge. Our results show that Isochlorogenic acid modulates the intestinal microbiota and elevates microbial diversity in lipopolysaccharide-challenged weaned lambs. Moreover, Isochlorogenic acid mitigates lipopolysaccharide-triggered intestinal injury by improving the antioxidant capacity of lambs.

## 1. Introduction

Early weaning was an efficient way to shorten the breeding cycles and enhance the efficiency of ewes in the lamb industry. However, early weaning also induced severe stress responses in lambs [1,2]. The combined stressors of maternal separation, dietary transition, and environmental alteration during the weaning period easily induce oxidative stress in lambs, further leading to hormonal imbalance, retarded growth, and increased diarrhoea incidence [3,4,5]. Li et al. previously demonstrated that early weaning disrupts intestinal microbiota composition and impairs intestinal barrier function-related gene expression in lambs, which is closely associated with weaning-induced growth inhibition and intestinal dysfunction [6]. Oxidative stress is defined as the imbalance between pro-oxidant and antioxidant systems, which triggers excessive accumulation of intestinal reactive oxygen species (ROS) and ultimately causes irreversible intestinal oxidative damage [7,8]. In contrast, inflammatory response is an endogenous defensive mechanism activated by tissue injury, pathogen infection, and external stimulation, functioning to eliminate harmful stimuli, repair damaged tissues, and restore physiological homeostasis.

Oxidative stress and inflammatory responses are two pivotal mechanisms underlying multiple physiological and pathological processes in animals [9]. In acute inflammatory states, massive neutrophil infiltration and excessive ROS production aggravate tissue oxidative damage, which in turn amplifies the inflammatory cascade and forms a mutually reinforcing pathological cycle [10]. Polyphenols are secondary metabolites widely distributed in dicotyledonous plants such as *Eucommia ulmoides*, *Stevia rebaudiana*, honeysuckle, and coffee, with prominent antioxidant biological activities [11]. In addition to antioxidant effects, plant polyphenols have attracted extensive research attention due to their potent anti-inflammatory, immunomodulatory, and antibacterial properties [12].

Isochlorogenic acid is a phenolic acid synthesized in plants through the shikimate pathway via the condensation of caffeic acid with quinic acid. It is a major byproduct during the industrial processing of *Stevia rebaudiana* sweeteners [13]. Structurally, ICGA is formed by the esterification of one quinic acid molecule with two caffeic acid molecules [14], and primarily consists of three active monomers, namely, isochlorogenic acid A, B, and C [15]. It has been elucidated that ICGA exerts diverse properties, including anti-inflammatory and antioxidative activities. ICGA relieved inflammatory reaction and oxidative stress via the down-regulation of NF-κB/NLRP3 signaling to treat LPS-induced acute lung injury [16]. ICGA protected cells from mycotoxin damage by maintaining mitochondrial dynamic homeostasis and improving innate immune function related to the RIG-I-like receptor (RLR) pathway [17]. Despite the well-documented antioxidant and anti-inflammatory activities of ICGA, the specific molecular mechanisms remain poorly elucidated. Furthermore, existing studies regarding ICGA are predominantly confined to disease models and monogastric animals, while relevant investigations in young ruminants are extremely limited. This study is the first to explore the application and protective mechanism of ICGA in young weaned lambs. Based on an LPS acute inflammatory challenge oxidative stress model, we evaluated the regulatory effects of dietary ICGA supplementation on immune function, antioxidant status, and intestinal barrier integrity in weaned lambs. This study provides novel theoretical evidence for the application of ICGA as a functional feed additive to improve intestinal health and relieve early weaned stress in young ruminant lambs.

## 2. Materials and Methods

### 2.1. Experimental Material

The ICGA (Lot No. 2-0969-240522) derived from Stevia is provided by Chenguang Biotechnology Co., Ltd. (Handan, China). The isochlorogenic acids (ICGA) from *Stevia rebaudiana* extract had a total effective content ≥ 60%, of which polyphenols account for 67.68%. Among the polyphenols, ICGA-A, ICGA-B, and ICGA-C account for 20.60%, 20.40%, and 23.20%, respectively, collectively representing 94.90% of the total polyphenol content [18]. LPS (O55:B5, #L2880) was purchased from Sigma-Aldrich (Saint Louis, MO, USA).

### 2.2. Ethical Considerations

These lambs were milk-reared suckling lambs with a well-defined genetic background, uniform rearing management, and consistent nutritional status prior to the experiment. All animals were reared under identical feeding and housing conditions to minimize confounding variables. Animal welfare information: Before tissue sampling, lambs were anesthetized by intravenous sodium pentobarbital from China Animal Husbandry Group (Beijing, China) (30 mg/kg body weight). Deep anesthesia was confirmed by loss of corneal and pedal withdrawal reflexes, followed by humane euthanasia via jugular exsanguination. Lambs were monitored continuously after intraperitoneal LPS injection until sampling, with records of rectal temperature, behaviour, feed intake, respiration and diarrhoea. The humane endpoints included severe depression, persistent recumbency, dyspnoea, body temperature > 42 °C or <37.5 °C, and inability to drink; immediate euthanasia would be performed once these signs appeared. No animals reached humane endpoints in this trial. LPS-challenged lambs only exhibited transient fever and mild depression, and there were no unexpected deaths. All procedures were approved by the Laboratory Animal Care Advisory Committee of Hebei Agricultural University (Approval No. 2023125).

### 2.3. Animals, Diets and Management

The animal experiment was carried out at Baosen Animal Husbandry Technology Co., Ltd. in Cangzhou, China (37°29′–38°57′ N, 115°42′–117°50′ E). Throughout the study period, the temperature was kept at 24–28 °C, and the mean relative humidity was kept at 45.3 ± 4.2%, which depended on natural ambient conditions, ranging from 41.0% to 50.5%. The experiment was a completely randomized design with forty-eight male Hu lambs (50 ± 2 days old, initial body weight of 15 ± 2.30 kg) being selected and randomly allocated into one of four treatments (12 lambs per treatment): control (CON), LPS, ICGA1, and ICGA2 groups. The lambs in the CON and LPS groups received a daily basal diet, while the lambs in the ICGA1 and ICGA2 groups received daily doses of 1.5 g/kg and 2.0 g/kg ICGA, respectively. The supplemental levels of ICGA were selected based on the findings of Li et al. [19]. At the end of the trial (on day 15), intestinal barrier injury was induced by intraperitoneal injection of LPS (200 μg/kg body weight), according to the method described by Wang et al. [20]. Preliminary experiments showed that 100 μg/kg only induced mild inflammation, whereas 200 μg/kg consistently caused significant intestinal oxidative injury and upregulated the TLR4/NF-κB pathway with no mortality in lambs. This dose is suitable for evaluating the protective effect of ICGA. The basal ration was prepared based on National Research Council (2007) recommendations [21]. The ingredients and nutritional composition of the basal ration were shown in Table 1. The experiment lasted 22 d, with 7 days of adaptation and 15 d of this study.

All the lambs were given ad libitum access to their respective diet and fresh water in individual pens (Each pen measured 0.8 m × 1.2 m). The experimental diet was separated into three equal parts and fed to the lambs at 06:00, 14:00, and 18:00 every day. The daily feed allowance for lambs was determined based on ad libitum feed intake recorded during the adaptation period, such that feed refusals accounted for 3–5% of the provided feed. The lambs were dewormed, ear-tagged, and vaccinated during adaptation.

### 2.4. Sampling and Measurement Requirements

All measurements and histological evaluations were conducted in a blinded fashion. Tissue sections were labelled with serial numbers only, and morphological measurements were carried out by a researcher who was not informed of treatment allocation to reduce observer bias. Five fields of view were randomly selected for measurement of villus height and crypt depth. A total of 10 villi and 5 crypts were measured across the five fields. The analysis was performed using SlideViewer 2.6 (3DHISTECH Ltd., Budapest, Hungary). A total of 48 weaned male lambs were selected by referencing published studies on intestinal stress in lambs to achieve adequate statistical power. Enrolled lambs were clinically healthy, with comparable age and body weight and no previous disease history. Any animals were excluded from the final dataset when they became sick, sustained injury, or showed abnormal feeding behaviour during the trial.

### 2.5. Growth Performance

During the experimental period, the daily basal diet intake of each lamb was recorded individually. Lambs were fasted and weighed before morning feeding on days 0 and 14 of the experiment. Average daily feed intake (ADFI), average daily gain (ADG), and feed conversion ratio (FCR) were calculated for each lamb.

### 2.6. Sample Collection and Processing

At the end of the trial, five lambs were randomly selected from each group after 4 h of LPS-challenged treatment on day 15 of the trial. Blood samples were collected from the jugular vein into 5 mL clot activator tubes from Hebei Kangweishi Medical Technology Co., Ltd. (Shijiazhuang, China), and samples were centrifuged at 3000 r/min for 10 min to obtain serum, which was stored at −20 °C until analysis. Ileum tissue and its digesta were collected for determination of related indicators. Approximately 2 cm of ileal tissue was fixed in 4% paraformaldehyde from Wuhan Servicebio Technology Co., Ltd. (Wuhan, China) for histopathological analysis. The remaining ileal tissue was stored at −80 °C for subsequent gene expression analysis, and the digesta were stored at −80 °C for microbial community analysis.

### 2.7. Serum Inflammatory and Antioxidative Stress Indexes

The serum concentrations of diamine oxidase (DAO), D-lactate (D-LA), endotoxin (ET), cortisol (COR), interleukin-1β (IL-1β), interleukin-6 (IL-6), and tumor necrosis factor-α (TNF-α) were quantified using commercial ELISA kits (Jiangsu Meimian Industrial Co., Ltd., Yancheng, China). Serum total antioxidant capacity (T-AOC), catalase (CAT), superoxide dismutase (SOD), glutathione peroxidase (GSH-Px), and malondialdehyde (MDA) were measured using biomarker assay kits from Nanjing Jiancheng Bioengineering Institute (Nanjing, China).

### 2.8. Histology of the Ileum Tissue

Ileal tissue samples were fixed in 4% paraformaldehyde. The tissue segments were dehydrated with ethanol, embedded in paraffin, and sectioned at 4–6 μm. Hematoxylin and eosin (H&E) staining was performed on the tissue slices, and ileal morphological structures were observed under a light microscope (Olympus, DP-26, Tokyo, Japan). The ileal villus height and crypt depth were measured.

### 2.9. Quantitative Real-Time PCR

Total RNA of the ileum tissue was extracted according to the manufacturer’s instructions and supplied with the Trizol^TM^ Reagent (Invitrogen, Carlsbad, CA, USA). The total RNA (1 μg) was reverse-transcribed into cDNA according to the instructions of the PrimeScript RT Reagent Kit with gDNA Eraser (Perfect Real Time, Takara, Otsu, Japan). The reverse transcription reaction was performed using a kit (Hangzhou Bioer Technology Co., Ltd., Hangzhou, China, BSB40M1), following the manufacturer’s instructions. The 20 μL reverse transcription system contained 10 μL Hybrid mix (contains dNTP, oligo dT and random primers, 1 μL enzyme mix, 2 μL of template RNA, and RNase-free dH_2_O up to 20 μL. Primers used for qPCR were designed and synthesized by Wuhan Servicebio Technology Co., Ltd. (Wuhan, China), which are listed in Table 2. Real-time quantitative PCR was performed using an iTaq Universal SYBR Green Supermix Kit (Bio-Rad Laboratories Inc., Hercules, CA, USA) on a real-time PCR instrument (LineGene 9600 Plus, Hangzhou Bori Biotechnology Co., Ltd., Hangzhou, China). The qPCR system included 10 μL of 2 × SYBR Green Mix, 0.4 μL of PCR-forward primer F, 0.4 μL of PCR-reverse primer R, 1 μL of cDNA, and RNase-free dH_2_O up to 20 μL. The amplification cycling conditions were as follows: 95 °C for 5 min, followed by 40 cycles of 95 °C for 15 s and 60 °C for 40 s. After the reaction, data were collected. Each sample was run in triplicate. Data analyses were performed according to the 2^−ΔΔCt^ method as described previously. *GAPDH* was used as a reference gene for the relative quantification real-time PCR experiments. The amplification efficiency of each primer pair was determined using melting curve analysis for all genes in the range of from 60 to 95 °C. The amplification efficiencies ranged from 90% to 110%. RNA integrity was assessed by agarose gel electrophoresis and RNA integrity number (RIN); all RNA samples had RIN > 7.0. The stability of *GAPDH* across treatments was evaluated using the ΔCt method; no significant difference in *GAPDH* Ct values was observed among groups, confirming its suitability as the reference gene.

### 2.10. DNA Extraction and High-Throughput Sequencing

The methods for analyzing the microbial diversity were performed according to Guo et al. [22]. Total genomic DNA was extracted from ileal digesta using the TGuide S96 Magnetic Soil/Fecal DNA Kit (Tiangen Biotech (Beijing) Co., Ltd., Beijing, China). DNA concentration and purity were determined with a NanoDrop 2000 spectrophotometer (Thermo Fisher Scientific, Waltham, MA, USA). The V3-V4 hypervariable region of the bacterial 16S rRNA gene was amplified using universal primers 338F (5′-ACTCCTACGGGAGGCAGCA-3′) and 806R (5′-GGACTACHVGGGTWTCTAAT-3′). Illumina index sequences were added to the 5′ ends of both forward and reverse primers for high-throughput sequencing. PCR amplification was performed using Phusion^®^ High-Fidelity PCR Master Mix with GC Buffer (New England Biolabs, Ipswich, MA, USA), and sterile water served as the PCR negative control. The thermal profile was as follows: initial denaturation at 95 °C for 5 min; 30 cycles of denaturation at 95 °C for 1 min, annealing at 60 °C for 1 min, and extension at 72 °C for 1 min; followed by a final extension at 72 °C for 7 min. Barcoded amplicons were pooled at equal molar concentrations for Illumina paired-end library construction and cluster generation, and sequencing was performed on the Illumina MiSeq platform. High-quality reads were clustered into operational taxonomic units (OTUs) at a 97% sequence similarity threshold using USEARCH v10.0. QIIME2 was employed to calculate α-diversity indices for assessing species richness and complexity within individual samples. Principal coordinate analysis (PCoA) based on β-diversity metrics was performed to assess differences in microbial community composition among groups. One-way analysis of variance (ANOVA) was used to compare bacterial relative abundances and α-diversity indices among treatments. Raw sequencing data have been deposited in the NCBI Sequence Read Archive (SRA) under accession number PRJNA1534686.

### 2.11. Statistical Analysis

For each indicator, measurements were performed in triplicate as technical replicates. Before statistical analysis, the normality of residuals and homogeneity of variance for all endpoints were checked using SPSS software version 22.0 (SPSS Inc., Chicago, IL, USA). One-way analysis of variance (ANOVA) was performed, followed by Tukey’s test for pairwise multiple comparisons among treatment groups. Linear and quadratic polynomial contrasts were not conducted in this study. Pearson correlation analysis was used for correlation analysis, and the false discovery rate (FDR, Benjamini-Hochberg method) was applied to correct for multiple testing. Differences were considered significant at *p* < 0.05.

## 3. Results

### 3.1. Effects of ICGA Pre-Challenge Feeding Response on Growth Performance

**Figure 1:**
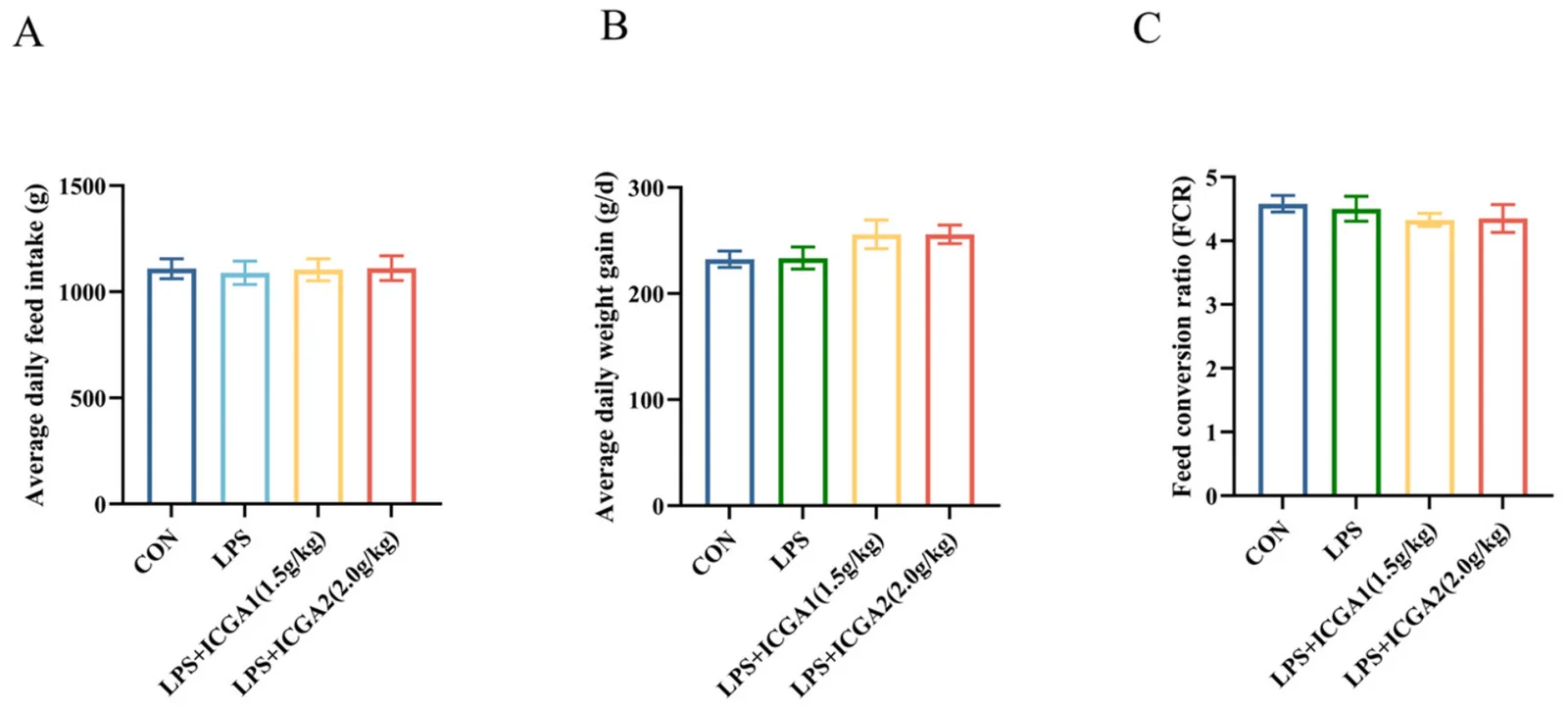
Effects of ICGA on the average daily feed intake (**A**), average daily gain (**B**), and feed conversion ratio (**C**) responses to acute LPS challenge in weaned lambs. CON, control group (basal diet); LPS, LPS-challenged lambs fed the basal diet; ICGA1, LPS-challenged lambs fed basal diet supplemented with 1.5 g/kg ICGA; ICGA2, LPS-challenged lambs fed basal diet supplemented with 2.0 g/kg ICGA. Twelve replicates were used for each indicator. Means with different superscript letters differ significantly (*p* < 0.05). Blue, CON group; Green, LPS group; Yellow, ICGA1 group; Red, ICGA2 group.

Compared with the CON group, lambs in the LPS-challenged group exhibited reduced appetite and lethargy after intraperitoneal LPS administration, but their ADFI did not differ significantly from that of the CON group (*p* > 0.05, Figure 1A). Lambs in the ICGA group had a higher body weight than those in the CON and LPS groups, but the difference was not statistically significant (*p* > 0.05, Figure 1B). No significant difference in feed conversion ratio (FCR) was observed among the CON, LPS-challenged, and ICGA groups (*p* > 0.05, Figure 1C).

### 3.2. Effects of ICGA on Serum Immune Parameters in LPS Lamb

**Figure 2:**
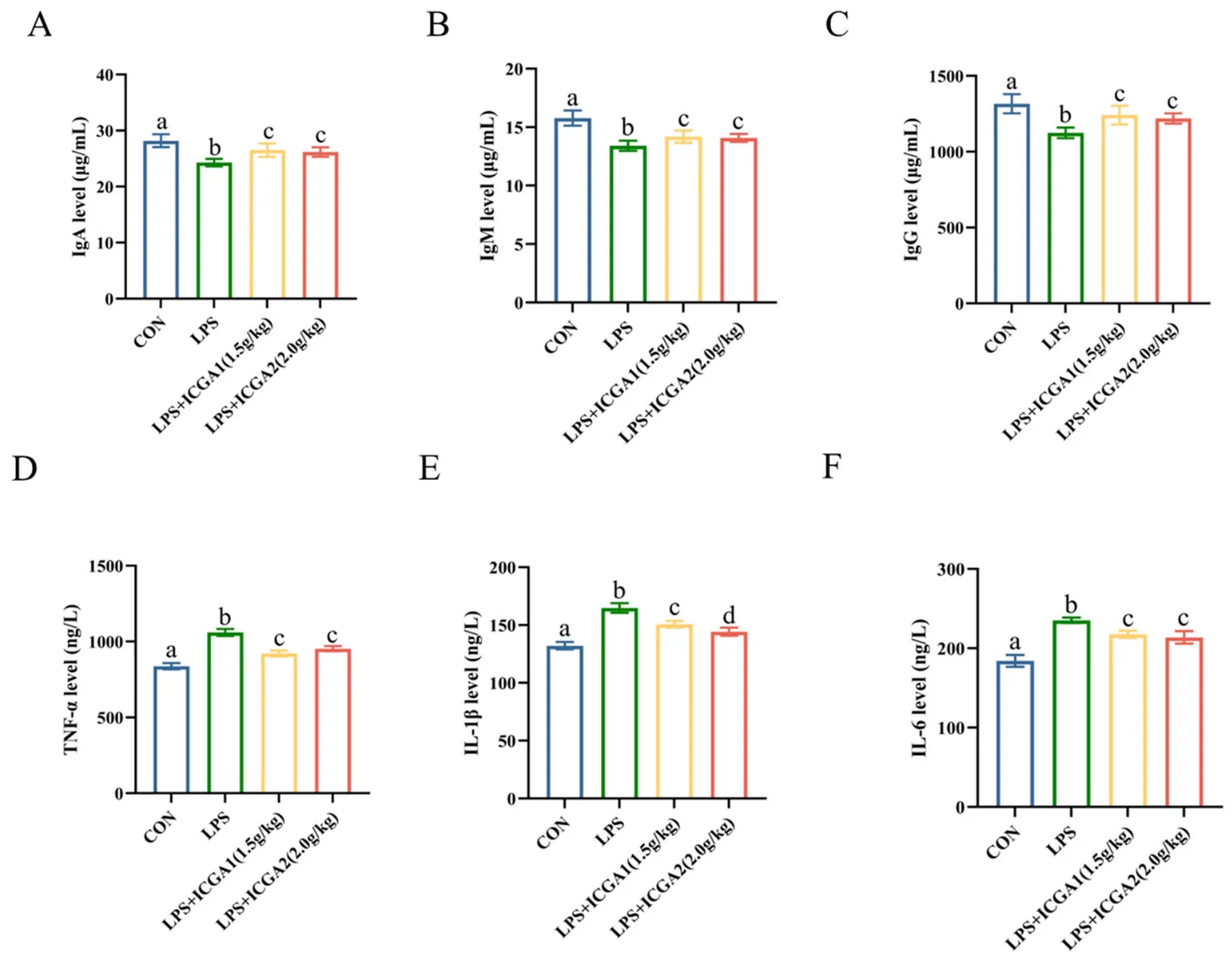
Effects of dietary supplementation of ICGA on serum immune parameters in LPS-challenged lambs. (**A**–**F**): Serum concentrations of IgA, IgM, IgG, TNF-α, IL-1β, and IL-6. CON, control group fed basal diet; LPS, LPS-challenged lamb fed the basal diet; ICGA1, LPS-challenged lambs fed basal diet supplemented with 1.5 g/kg ICGA; ICGA2, LPS-challenged lambs fed basal diet supplemented with 2.0 g/kg ICGA. Five replicates were used for each indicator. Values with different lowercase superscript letters differ significantly (*p* < 0.05). Blue, CON group; Green, LPS group; Yellow, ICGA1 group; Red, ICGA2 group.

The results showed that LPS significantly decreased the levels of IgA, IgM, and IgG (*p* < 0.05, Figure 2A–C), and increased serum levels of TNF-α, IL-1β, and IL-6 (*p* < 0.05, Figure 2D–F) compared with the CON group. Compared with the LPS group, the serum concentration of TNF-α, IL-1β, and IL-6 in ICGA groups significantly decreased (*p* < 0.05, Figure 2D–F), and the levels of IgA, IgG, and IgM were increased significantly (*p* < 0.05, Figure 2A–C). However, there was no significant difference in the concentration of serum immune indices in the ICGA1 and ICGA2 groups, except for IL-1β (*p >* 0.05, Figure 2A–D,F). The results indicated that ICGA intervention could decrease the levels of inflammatory factors in LPS lambs.

### 3.3. Effects of ICGA on the Serum Oxidative Indices in LPS Lamb

**Figure 3:**
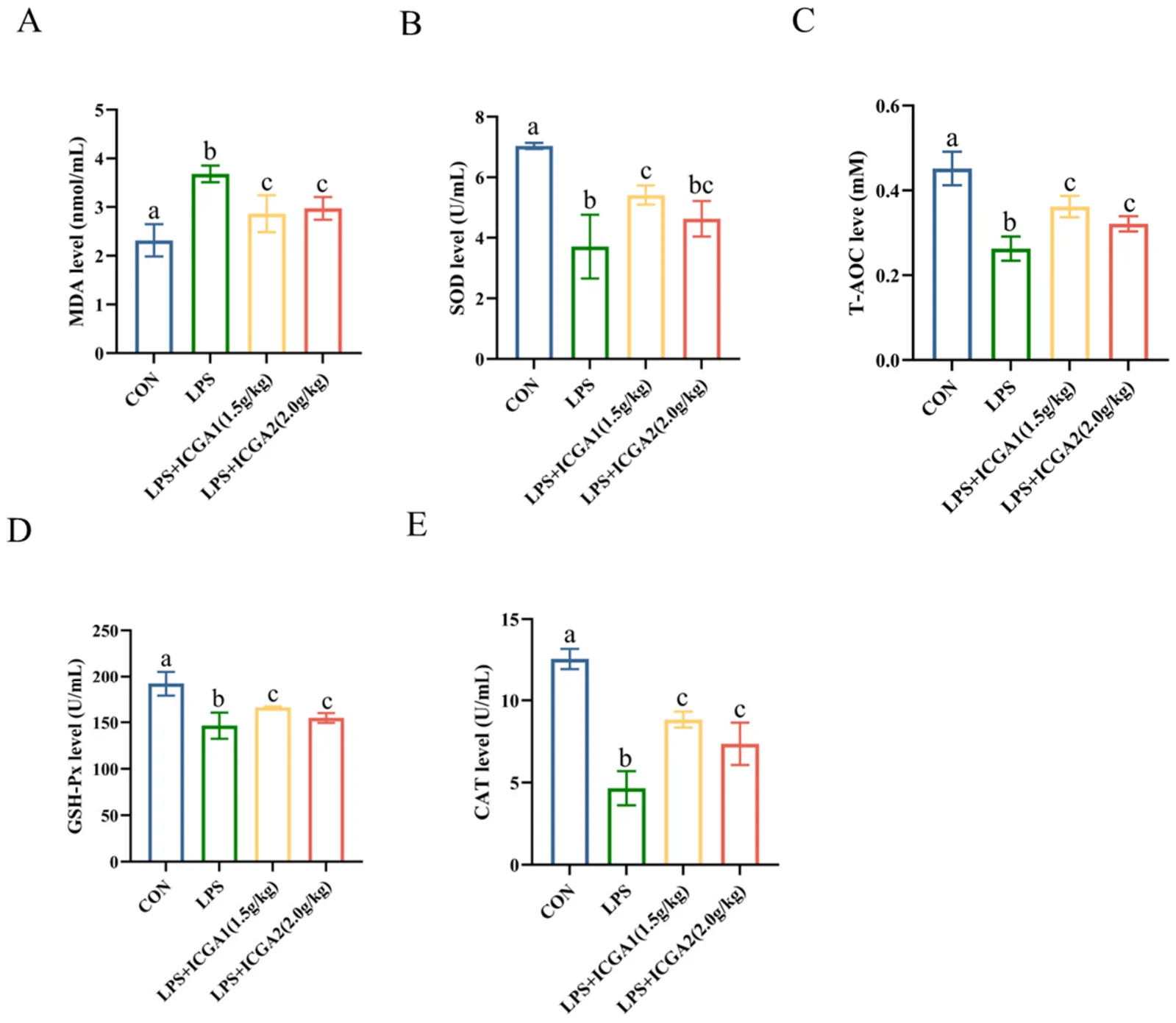
Effects of dietary supplementation of ICGA on serum oxidative indices in LPS-challenged lambs. (**A**–**E**) Serum concentrations of MDA, SOD, T-AOC, GSH-Px, and CAT. CON, control group fed basal diet; LPS, LPS-challenged lambs fed the basal diet; ICGA1, LPS-challenged lambs fed basal diet supplemented with 1.5 g/kg ICGA; ICGA2, LPS-challenged lambs fed basal diet supplemented with 2.0 g/kg ICGA. Five replicates were used for each indicator. Values with different lowercase superscript letters differ significantly (*p* < 0.05). Blue, CON group; Green, LPS group; Yellow, ICGA1 group; Red, ICGA2 group.

As shown in Figure 3, compared with the CON group, the concentration of SOD, T-AOC, GSH-Px, and CAT decreased significantly (*p* < 0.05, Figure 3B–E) in the LPS-challenged group; however, the MDA level was significantly increased (*p* < 0.05, Figure 3A). In contrast, compared with the LPS-challenged group, the T-AOC, GSH-Px, and CAT concentrations increased significantly (*p* < 0.05, Figure 3C–E), and the MDA levels decreased (*p* < 0.05, Figure 3A) in ICGA groups.

### 3.4. Effects of ICGA on the Serum Intestinal Injury in LPS Lamb

**Figure 4:**
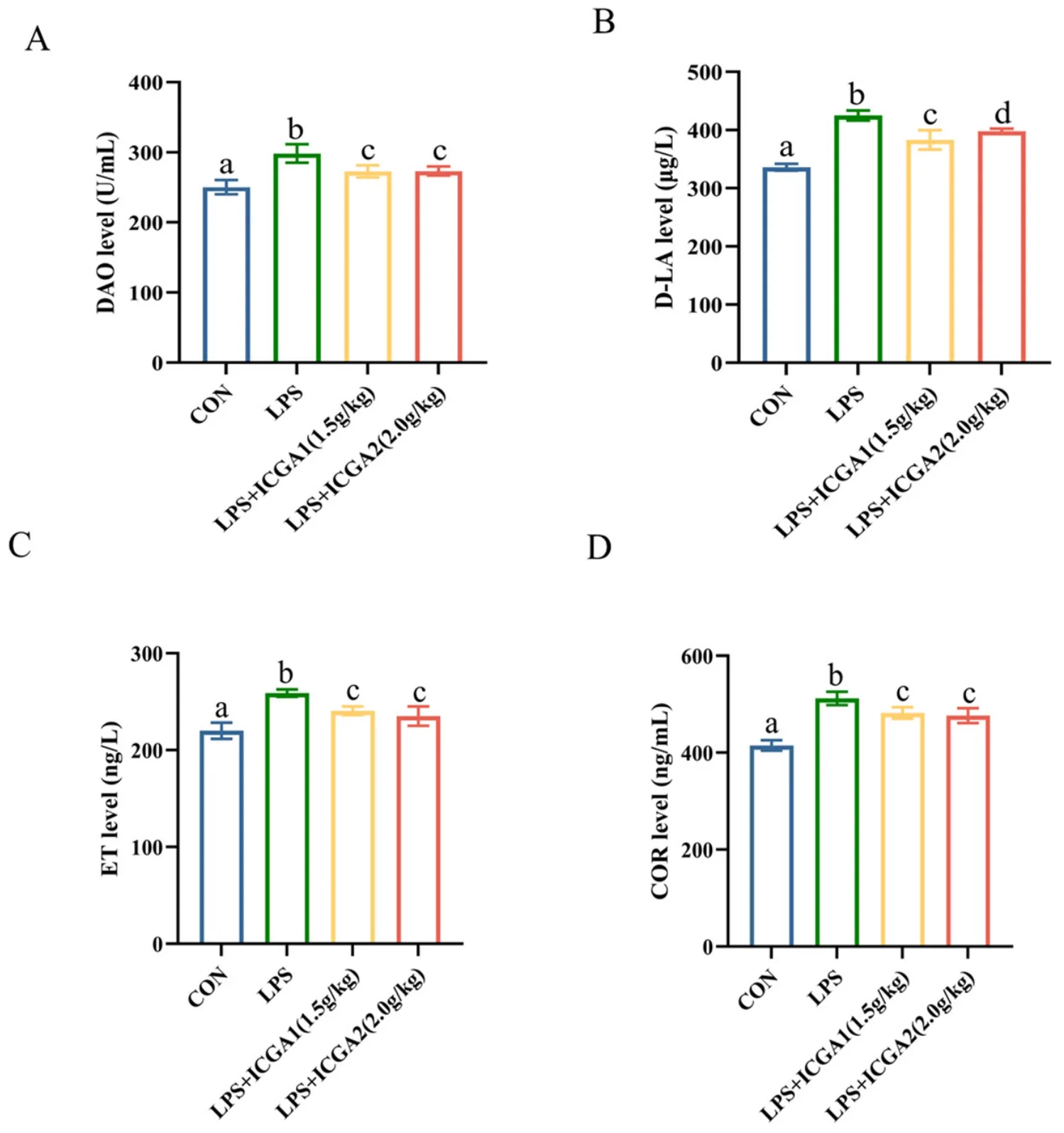
Effects of dietary supplementation of ICGA on serum intestinal injury indices in LPS-challenged lambs. (**A**–**D**) Serum concentrations of DAO, D-LA, ET, and COR. CON, control group fed basal diet; LPS, LPS-challenged lambs fed the basal diet; ICGA1, LPS-challenged lambs fed basal diet supplemented with 1.5 g/kg ICGA; ICGA2, LPS-challenged lambs fed basal diet supplemented with 2.0 g/kg ICGA. Five replicates were used for each indicator. Values with different lowercase superscript letters differ significantly (*p* < 0.05). Blue, CON group; Green, LPS group; Yellow, ICGA1 group; Red, ICGA2 group.

Compared with the CON group, the DAO, D-LA, ET, and COR levels increased significantly (*p* < 0.05, Figure 4A–D) in the LPS group. In contrast, the DAO, D-LA, ET, and COR concentrations decreased significantly (*p* < 0.05, Figure 4A–D) in the ICGA groups compared with the LPS-challenged group. The D-LA level was higher (*p* < 0.05, Figure 4B) in the ICGA2 group than in the ICGA1 group.

### 3.5. Effects of ICGA on the Morphology Indices of Ileum in LPS Lambs

**Figure 5:**
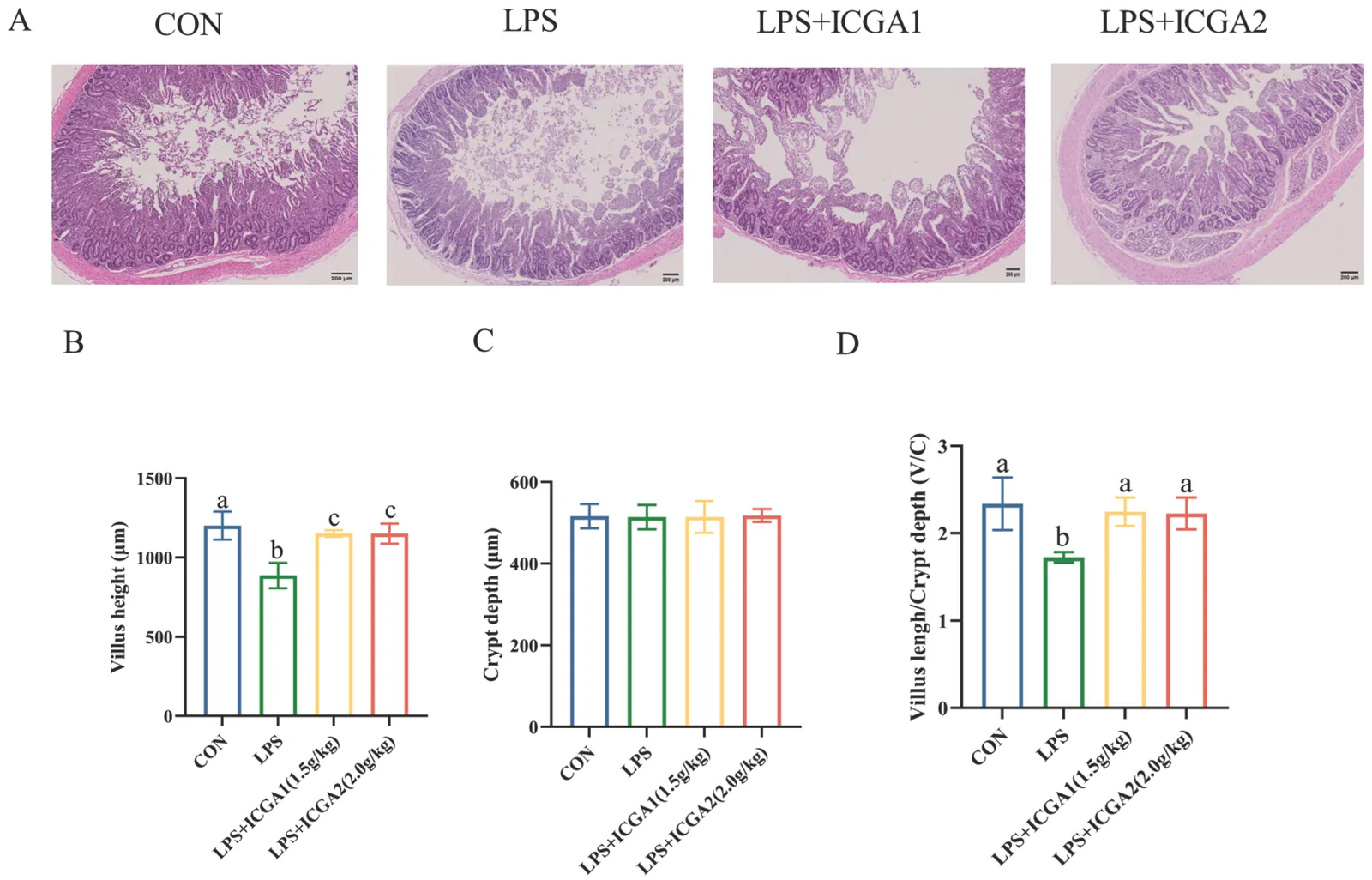
Effects of dietary supplementation of ICGA on the ileal morphology (**A**) and indices (**B**–**D**) in LPS-challenged lambs. CON, control group fed basal diet; LPS, LPS-challenged lambs fed the basal diet; ICGA1, LPS-challenged lambs fed basal diet supplemented with 1.5 g/kg ICGA; ICGA2, LPS-challenged lambs fed basal diet supplemented with 2.0 g/kg ICGA. Five replicates were used for each indicator. Values with different lowercase superscript letters differ significantly (*p* < 0.05). Blue, CON group; Green, LPS group; Yellow, ICGA1 group; Red, ICGA2 group. Scale: 200 μm.

Histopathological observation of lamb ileal sections showed that the CON group had intact intestinal mucosa, with closely and regularly aligned villi and normal epithelial cell morphology (Figure 5A). In contrast, the LPS-challenged group exhibited typical intestinal pathological lesions, including shortened villi, partial shedding of villous epithelium, and loss of goblet cells (Figure 5A). In the ICGA treatment groups, epithelial exfoliation was markedly attenuated, villus arrangement remained orderly, inflammatory cell infiltration was reduced, and intestinal inflammatory injury was effectively alleviated (Figure 5A).

Quantitative analysis of ileal morphological parameters, including villus height and crypt depth, was further performed. The ileal villus height was lower in the LPS-challenged group than that in the CON group (*p* < 0.05, Figure 5B). Compared with the LPS group, lambs in the ICGA groups exhibited significantly greater ileal villus height (*p* < 0.05, Figure 5B). However, statistical analysis revealed no significant difference in crypt depth across all treatment groups (*p* > 0.05, Figure 5C). Additionally, the villus height/crypt depth (V/C) ratio was decreased in the LPS-challenged group relative to the CON group (*p* < 0.05, Figure 5D), and the ICGA groups had a significantly higher V/C ratio compared with the LPS-challenged group (*p* < 0.05, Figure 5D). There was no significant difference between the ICGA1 and ICGA2 groups for ileal villus height (*p* > 0.05, Figure 5B) and V/C (*p* > 0.05, Figure 5D).

### 3.6. Effects of ICGA on the Barrier-Related Gene Expression of Ileum in LPS Lamb

**Figure 6:**
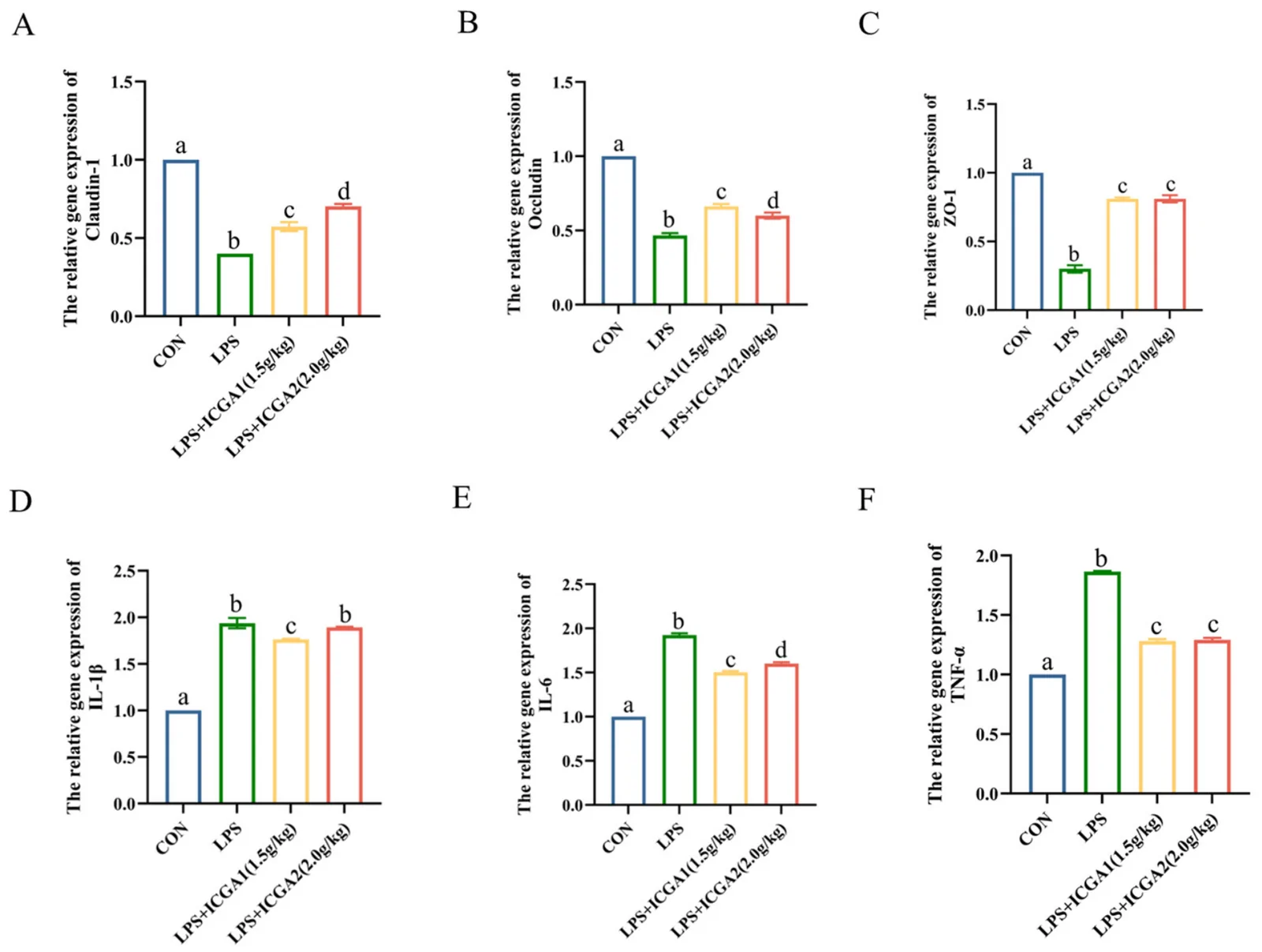
Effects of ICGA on the expression of ileal inflammatory factors and tight junction proteins. (**A**–**C**): The relative mRNA expression of Claudin-1, Occludin, and ZO-1; (**D**–**F**): The relative mRNA expression of IL-1β, IL-6, TNF-α; CON, control group fed basal diet; LPS, LPS-challenged lambs fed the basal diet; ICGA1, LPS-challenged lambs fed basal diet supplemented with 1.5 g/kg ICGA; ICGA2, LPS-challenged lambs fed basal diet supplemented with 2.0 g/kg ICGA. Five replicates were used for each indicator. Values with different lowercase superscript letters differ significantly (*p* < 0.05). Blue, CON group; Green, LPS group; Yellow, ICGA1 group; Red, ICGA2 group.

The relative mRNA expression of *Claudin-1*, *Occludin*, and *ZO-1* was significantly decreased in the LPS-challenged group compared with the CON group (*p* < 0.05, Figure 6A–C). However, there was significantly upregulated mRNA expression of *Claudin-1*, *Occludin*, and *ZO-1* in the ICGA1 and ICGA2 groups compared with the LPS-challenged group (*p* < 0.05, Figure 6A–C). *Claudin-1* mRNA expression was significantly higher (*p* < 0.05, Figure 6A) in the ICGA2 group than in the ICGA1 group. In addition, the *Occludin* mRNA expression was significantly decreased (*p* < 0.05, Figure 6B) in the ICGA2 group relative to the ICGA1 group. Additionally, the mRNA expression of IL-1β, IL-6, and TNF-α was significantly increased in the LSP-challenged group compared with the CON group (*p* < 0.05, Figure 6D–F). Supplementation with ICGA significantly reduced the mRNA expression of IL-1β, IL-6, and TNF-α (*p* < 0.05, Figure 6D–F).

### 3.7. Effects of ICGA on the TLR4/NF-kB/MyD88 Signaling Pathway of Ileum in LPS Lamb

**Figure 7:**
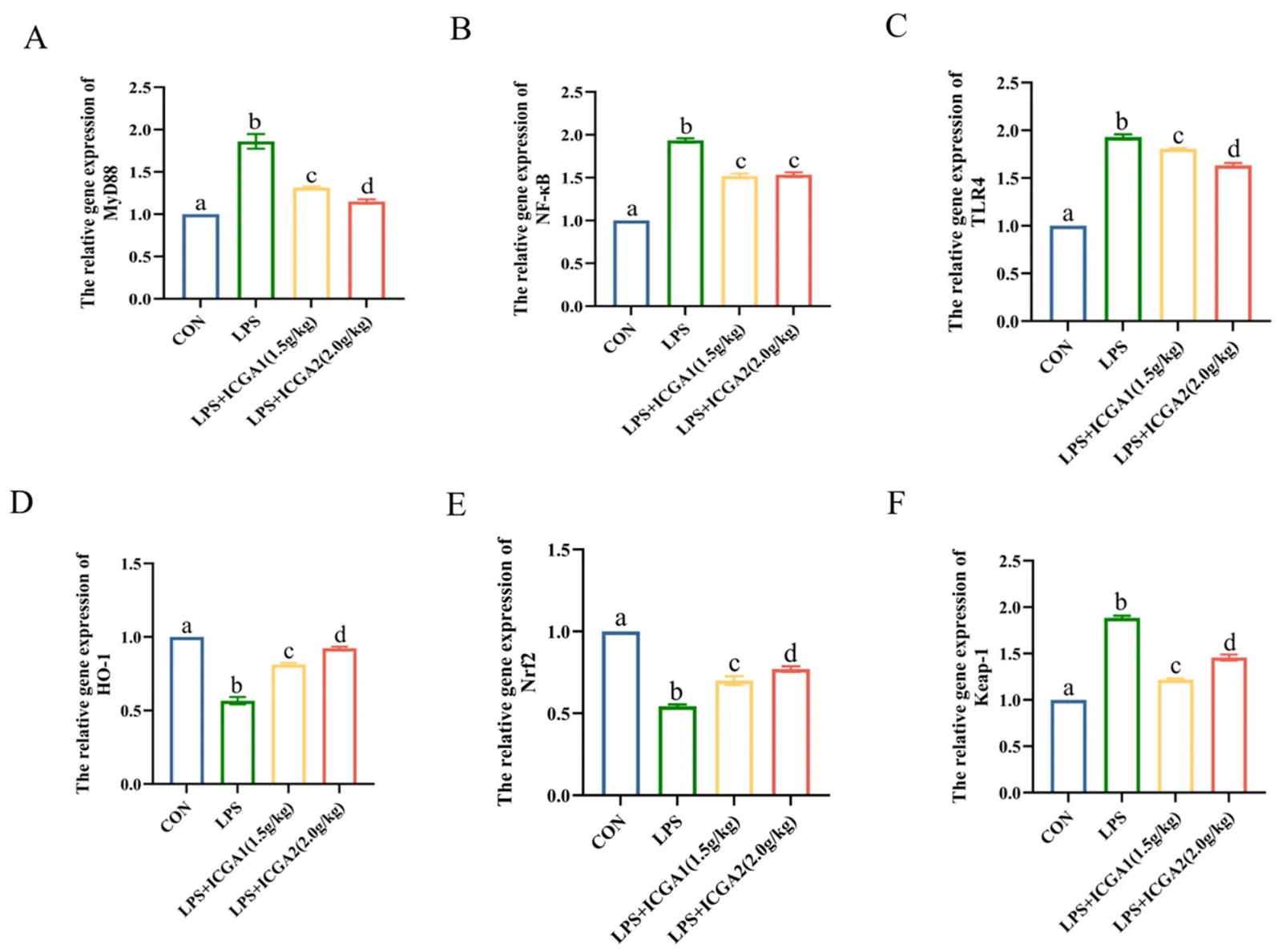
Effects of ICGA on the relative mRNA expression of TLR4/NF-κB and Nrf2/HO-1 signaling pathways in the ileum. (**A**–**F**) The relative mRNA expression of MyD88, NF-κB, TLR4, HO-1, Nrf2 and Keap1. CON, control group fed basal diet; LPS, LPS-challenged lambs fed the basal diet; ICGA1, LPS-challenged lambs fed basal diet supplemented with 1.5 g/kg ICGA; ICGA2, LPS-challenged lambs fed basal diet supplemented with 2.0 g/kg ICGA. Five replicates were used for each indicator. Values with different lowercase superscript letters differ significantly (*p* < 0.05). Blue, CON group; Green, LPS group; Yellow, ICGA1 group; Red, ICGA2 group.

The gene expression of MyD88, NF-κB, and TLR4 (*p* < 0.05, Figure 7A–C) was significantly increased in the LPS-challenged group compared with the CON group. In addition, compared with the LPS-challenged group, ICGA supplementation markedly reduced the relative mRNA expression of ileal MyD88, NF-κB, and TLR4 (*p* < 0.05, Figure 7A–C). Meanwhile, the gene expression levels of Nrf2 (*p* < 0.05, Figure 7E) and HO-1 (*p* < 0.05, Figure 7D) were significantly higher; however, the keap1 gene expression (*p* < 0.05, Figure 7F) showed lower expression in the ICGA1 and ICGA2 groups than in the LPS-challenged group. Furthermore, the relative mRNA expression of TLR4 (*p* < 0.05, Figure 7C) and MyD88 (*p* < 0.05, Figure 7A) were significantly decreased in the ICGA2 group compared with the ICGA1 group. However, compared with the ICGA1 group, there was a significant increase in the mRNA expression of HO-1, Nrf2, and keap1 in the ICGA2 group (*p* < 0.05, Figure 7D–F).

### 3.8. Effects of ICGA on Ileal Microbial Community Abundance

**Figure 8:**
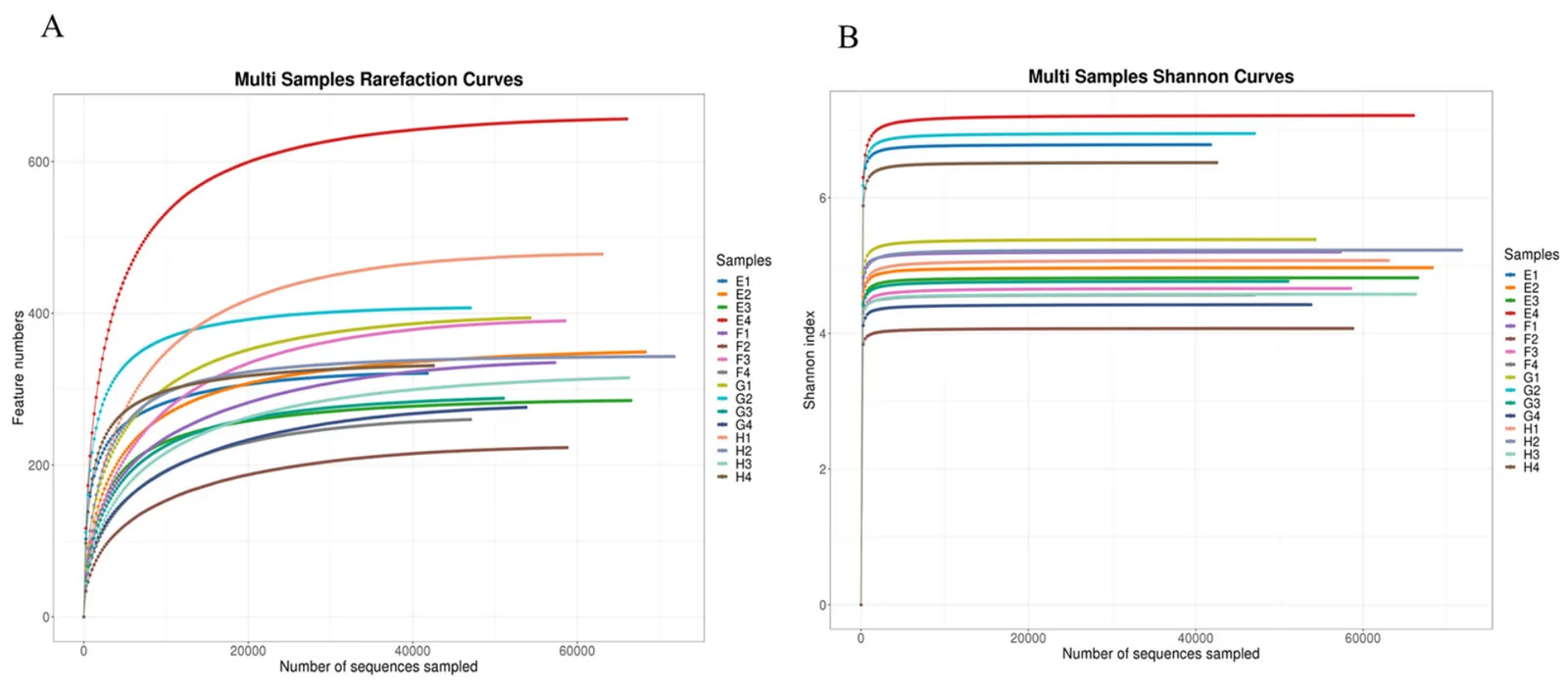
Sample rarefaction curves. (**A**) OTU number dilution curve; (**B**) Shannon index dilution curve. CON, control group fed basal diet, E1-E4; LPS, LPS-challenged lambs fed the basal diet, F1-F4; ICGA1, LPS-challenged lambs fed basal diet supplemented with 1.5 g/kg ICGA, G1-G4; ICGA2, LPS-challenged lambs fed basal diet supplemented with 2.0 g/kg ICGA, H1-H4. Four replicates were used for each indicator.

For 16S rRNA gene sequencing, five samples per group were initially analyzed. Outliers were identified using a uniform, objective algorithm across all groups, rather than by arbitrary manual removal. One outlier per group was excluded, leaving four samples per group for the final analysis. The rarefaction curves of ileal digesta samples (*n* = 4 per group) from weaned lambs rose rapidly and then plateaued (Figure 8), indicating that the sequencing depth was adequate to capture microbial taxa within the samples and verifying the reliability of the sequencing data.

**Figure 9:**
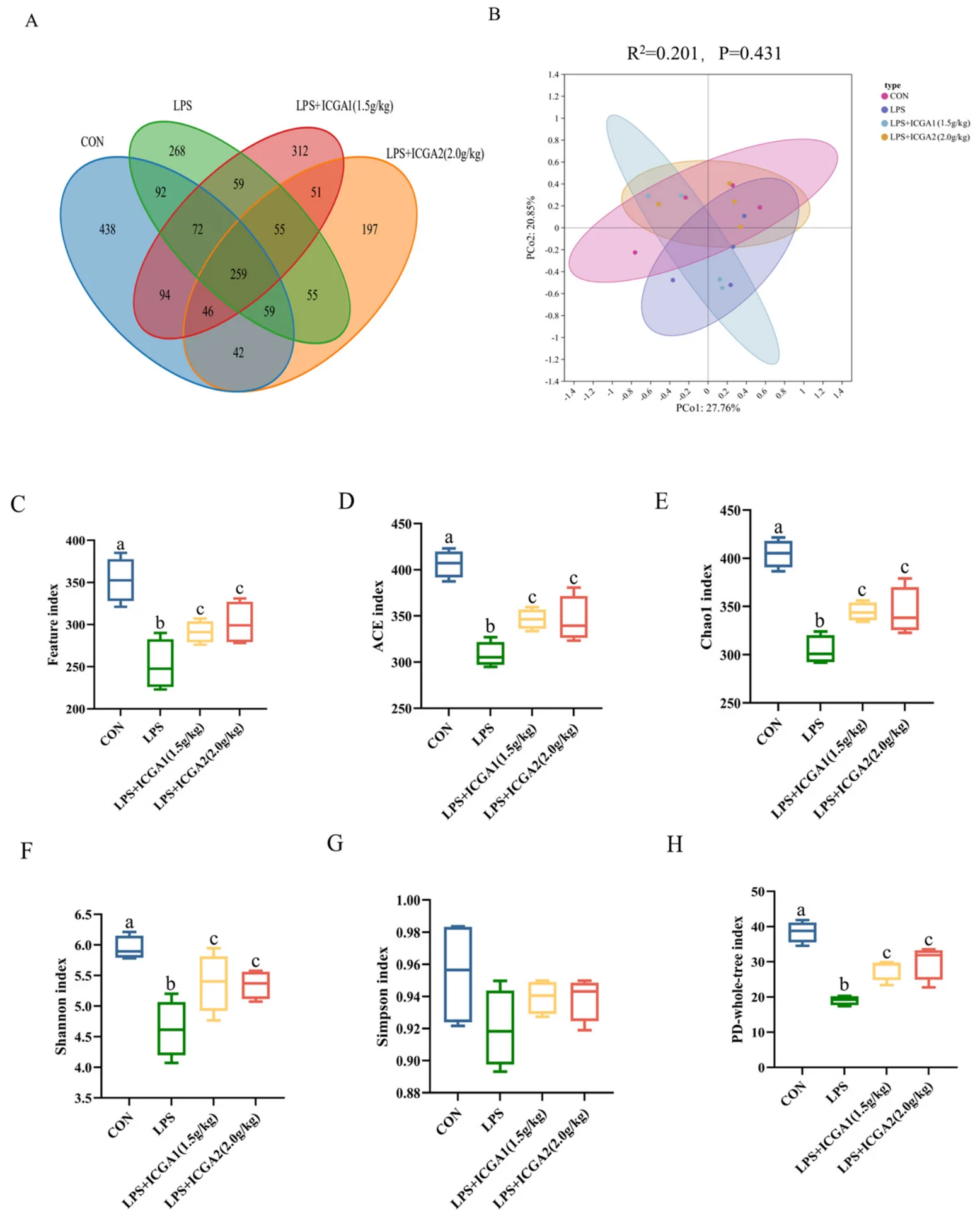
Effects of ICGA on ileal microbial diversity in LPS-challenged lambs. (**A**–**H**) Venn diagram, PCoA plot, Feature index, ACE index, Chao1 index, Shannon index, Simpson index, PD-whole-tree index. CON, control group fed basal diet; LPS, LPS-challenged lambs fed the basal diet; ICGA1, LPS-challenged lambs fed basal diet supplemented with 1.5 g/kg ICGA; ICGA2, LPS-challenged lambs fed basal diet supplemented with 2.0 g/kg ICGA. Four replicates were used for each indicator. Values with different lowercase superscript letters differ significantly (*p* < 0.05). Blue, CON group; Green, LPS group; Yellow, ICGA1 group; Red, ICGA2 group.

A total of 2099 operational taxonomic units (OTUs) were obtained from sequencing data. Principal coordinate analysis (PCoA) based on the binary-Jaccard algorithm revealed slight visual separation of microbial community structure among groups. However, PERMANOVA analysis further confirmed that the overall bacterial community composition did not differ significantly between treatments (R^2^ = 0.201, *p* = 0.431, Figure 9B). Alpha-diversity analysis showed that the Feature index, ACE, Chao1, Shannon, and PD-whole-tree indices in the lamb ileum were significantly decreased following LPS challenge (*p* < 0.05, Figure 9C–F,H). Dietary ICGA supplementation markedly reversed these LPS-challenged reductions in alpha-diversity indices (*p* < 0.05, Figure 9C–F,H). Principal coordinate analysis (PCoA, Figure 9B) based on the binary-Jaccard algorithm revealed distinct separation between the LPS group and the CON group (Figure 9A), while ICGA-supplemented groups were positioned closer to the CON group (*p* < 0.05, Figure 9C–F,H).

**Figure 10:**
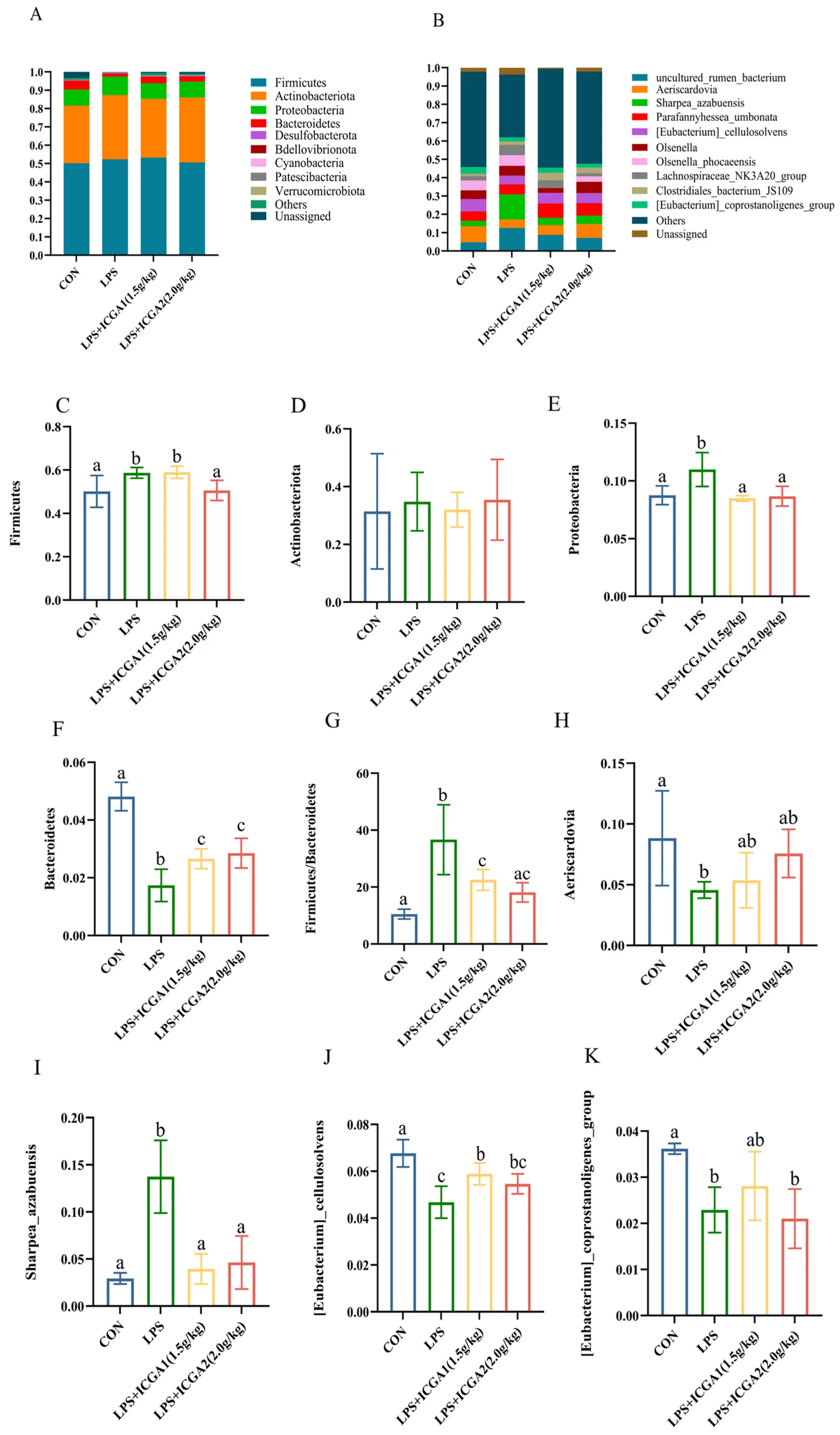
Effect of ICGA on ileal microbial community abundance. (**A**,**B**) Species accumulation curves at both genus and family levels, showing only the 10 most abundant species. (**C**–**G**) Relative abundance of *Firmicutes*, *Actinobacteriota*, *Proteobacteria*, *Bacteroidetes*, and *Firmicutes/Bacteroidetes*. (**H**–**K**): Relative abundance of *Aeriscardovia*, *Sharpea_azabuensis*, *[Eubacterium]_cellulosolvens*, *[Eubacterium]_coprostanoligenes*_group. CON, control group fed basal diet; LPS, LPS-challenged lambs fed the basal diet; ICGA1, LPS-challenged lambs fed basal diet supplemented with 1.5 g/kg ICGA; ICGA2, LPS-challenged lambs fed basal diet supplemented with 2.0 g/kg ICGA. Four replicates were used for each indicator. Values with different lowercase superscript letters differ significantly (*p* < 0.05). Blue, CON group; Green, LPS group; Yellow, ICGA1 group; Red, ICGA2 group. *p*-values were adjusted for multiple testing using the Benjamini–Hochberg false discovery rate (FDR) method.

Compared with the CON group, at the phylum level, the relative abundances of *Firmicutes* (Figure 10C), *Proteobacteria* (Figure 10E), and the *Firmicutes*/*Bacteroidetes* ratio (Figure 10G) were significantly increased in the LPS group (*p* < 0.05), whereas the relative abundance of *Bacteroidetes* was significantly decreased in the LPS-challenged group (*p* < 0.05, Figure 10F). The relative abundance of *Firmicutes* was significantly higher in the ICGA1 group than in the ICGA2 group (*p* < 0.05, Figure 10C). However, there were no significant differences in the relative abundances of *Proteobacteria* (Figure 10E), *Bacteroidetes* (Figure 10F) and the *Firmicutes*/*Bacteroidetes* ratio (Figure 10G) between the two ICGA-supplemented groups (*p* > 0.05).

At the genus level, the relative abundances of *Aeriscardovia* (Figure 10H), *[Eubacterium]_cellulosolvens* (Figure 10J) and *[Eubacterium]_coprostanoligenes*_group (Figure 10K) were markedly reduced (*p* < 0.05), while *Sharpea-azabuensis* was significantly elevated (*p* < 0.05, Figure 10I) in the LPS-challenged group compared with the other groups. There were no significant differences among the ICGA intervention groups for these three genera (*p* > 0.05, Figure 10H–K).

### 3.9. Correlation Between Intestinal Microorganisms and Inflammatory Factors, Antioxidant Indexes, and Intestinal Injury

**Figure 11:**
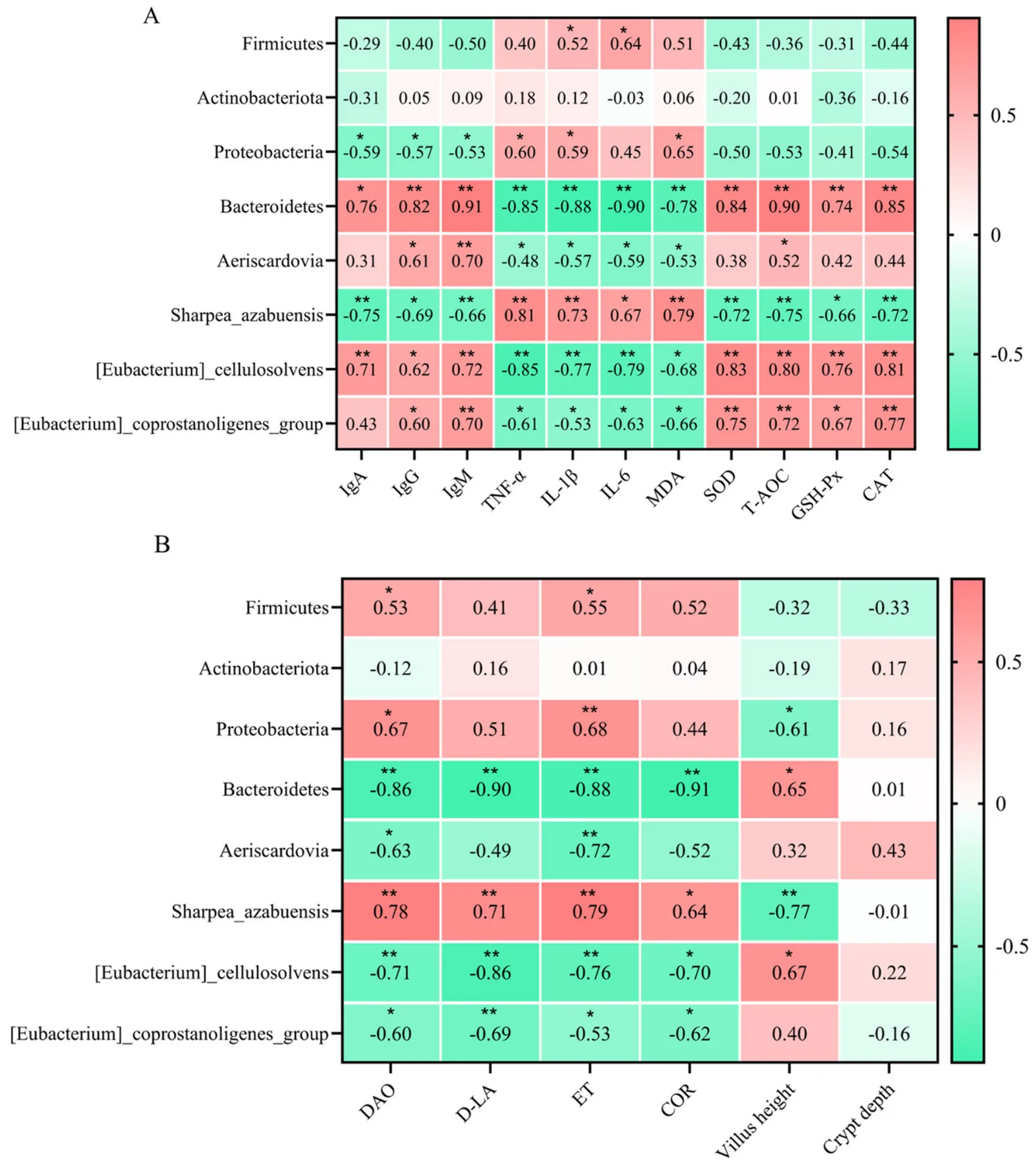
Correlation analysis of intestinal flora. * *p* < 0.05, ** *p* < 0.01. (**A**) Correlation analysis between intestinal microbiota and immune and antioxidant indices; (**B**) Correlation analysis between intestinal microbiota and intestinal injury indices, villus height, and crypt depth. Four replicates were used for each indicator. Pearson correlation analysis was performed using pooled data across all groups (*n* = 16). *p*-values were adjusted for multiple testing using the Benjamini–Hochberg false discovery rate (FDR) method.

The results showed that *Proteobacteria* and *Sharpea_azabuensis* were significantly positively correlated with inflammatory markers (IL-1β and IL-6, Figure 11A) and intestinal injury indicators (DAO and ET; *p* < 0.05, Figure 11B). *Bacteroidetes* and *[Eubacterium]_cellulosolvens* exhibited significant positive correlations with immunoglobulins (IgA, IgG, IgM; *p* < 0.05, Figure 11A) and antioxidant enzymes (SOD, T-AOC, GSH-Px, CAT; *p* < 0.05, Figure 11A). For *[Eubacterium]_coprostanoligenes_group*, significant positive correlations were observed with IgG, IgM, and antioxidant enzymes (SOD, T-AOC, GSH-Px, CAT; *p* < 0.05, Figure 11A), while no significant correlation was detected for IgA. Moreover, *Aeriscardovia* was significantly positively correlated with immunoglobulins (IgG, IgM) and T-AOC (*p* < 0.05, Figure 11A), while showing significant negative correlations with TNF-α, IL-1β, IL-6, MDA (*p* < 0.05, Figure 11A), and intestinal injury markers (DAO, ET; *p* < 0.05, Figure 11B). Additionally, *[Eubacterium]_cellulosolvens* and *Bacteroidetes* were significantly positively correlated with villus height (*p* < 0.05, Figure 11B).

## 4. Discussion

### 4.1. ICGA Improves the Growth of Lambs

Oxidative stress can induce intestinal dysfunction and impair gut health in animals during the breeding process, ultimately affecting growth performance [6]. Growth performance serves as the most intuitive indicator for evaluating the growth status of animals. Appropriate strategies to alleviate oxidative stress and enhance the growth of livestock and poultry are essential for improving economic returns in animal husbandry. Isochlorogenic acid (ICGA), a phenolic acid compound, is the primary active component of *Stevia rebaudiana* [23] and has strong anti-inflammatory, antioxidant, antibacterial, and immunomodulatory activities [15]. ICGA improves the digestibility of calcium and crude protein in broilers [24]. Ruminant studies have demonstrated that isochlorogenic acid modulates concentrations of butyric acid and total volatile fatty acids in ruminal fluid of sheep, thereby elevating dietary dry matter degradability and enhancing the feed conversion ratio [19]. Furthermore, ICGA confers protective effects against toxin infections, such as by attenuating the LPS-induced elevation of intestinal permeability and alleviating intestinal oxidative damage and inflammatory responses [25]. In this study, although no significant differences in growth performance were detected among groups, the ICGA pre-protection groups exhibited numerically higher ADG and ADFI relative to the CON and LPS-challenged groups. This numerical elevation suggested that pre-supplementation with ICGA tended to improve feed intake and daily weight gain under LPS acute stress. The lack of statistical significance may be partly attributed to the short experimental period and the acute LPS challenge applied only at the end of the trial. Collectively, ICGA exerted a mild beneficial trend on growth performance, while its prominent protective effects were mainly reflected in intestinal barrier function, antioxidant status, and gut microbiota rather than production performance.

### 4.2. ICGA Attenuates LPS-Induced Systemic Inflammation and Immune Suppression

The immune system serves as a vital defense mechanism against stress induced by various adverse environmental conditions, including heat or cold stress, weaned stress, and other stressors. Serum immunoglobulins (IgA, IgG, IgM) are key components of the immune system, and their concentrations represent crucial indicators of systemic immune function [26]. IgA specifically binds to antigens and eliminates them through precipitation and phagocytosis without causing inflammation [27]. IgG is the most abundant immunoglobulin in the body and plays a pivotal role in defending against microbial toxins. Meanwhile, IgM participates in the clearance of pathogenic bacteria, and IgG and IgM exert synergistic bactericidal and immune effects [28]. As a phenolic acid compound, ICGA has been reported to enhance immune function in poultry and livestock. Zhou et al. confirmed that serum IgG concentration exhibited a quadratic increase with increasing dietary ICGA levels [24]. Similarly, Huang et al. demonstrated that ICGA-A alleviates periodontitis by inhibiting inflammation [29].

Inflammation represents a protective pathological response of the organism to harmful stimuli. As a component of the outer membrane of Gram-negative bacteria composed of lipids and polysaccharides, LPS binds to cell-surface TLR4 receptors to activate the MyD88/NF-κB signaling pathway, which further triggers the synthesis and secretion of pro-inflammatory cytokines including IL-6, IL-1β, and TNF-α [30]. Excessive release of these cytokines substantially contributes to the initiation and progression of intestinal inflammation [31]. TNF-α participates in regulating intestinal permeability and is tightly related to the integrity of the intestinal mucosal barrier. Consistent with this mechanism, a rat diarrhea model observed marked upregulation of the gene and protein expression of TLR4, MyD88, and NF-κB in colonic tissues, which paralleled increased serum concentrations of IL-6 and TNF-α [32]. A study also showed that ICGA enhances host immune function through inhibition of the MyD88/NF-κB inflammatory cascade and activation of the Nrf2-dependent antioxidant signaling pathway [33]. In the present LPS-challenged lamb model, ICGA pre-supplementation downregulated the expression of inflammatory mediators, which may be partly attributed to the dual regulation of these two core signaling pathways.

Intestinal inflammation triggers a strong oxidative stress response and impairs cellular antioxidant capacity. Excessive free radicals induce lipid peroxidation to generate MDA, which further damages protein structure and function. SOD, GSH-Px, and CAT are the primary antioxidant enzymes in the organism, and they scavenge free radicals and suppress peroxide formation. As an active natural phenolic compound extracted from *Stevia rebaudiana*, ICGA alleviates LPS-induced oxidative stress by increasing the activities of SOD, GSH-Px, and CAT while reducing MDA concentration. Meanwhile, the gene expression of HO-1 and Nrf2 was significantly upregulated. Nrf2 serves as the master regulator of the oxidative stress response, and activated Nrf2 initiates downstream antioxidant cascades [34]. Under physiological conditions, Nrf2 binds to Keap1 and remains transcriptionally silent. Oxidative stress promotes the dissociation of Nrf2 from Keap1; subsequently, Nrf2 translocates into the nucleus and induces the expression of antioxidant genes, including HO-1 [35]. As a critical downstream effector of the Nrf2 signaling pathway, HO-1 exerts prominent anti-inflammatory and antioxidant functions. Chen et al. reported that tea polyphenol supplementation activated the Nrf2-ARE signaling pathway, which was linked to improvements in antioxidant and immune responses [36]. These findings indicate that ICGA enhances the antioxidant capacity of lambs and mitigates LPS-challenged oxidative injury, concomitant with altered expression of Nrf2-related genes.

### 4.3. ICGA Intestinal Permeability and Morphology of Lambs

Oxidative stress is one of the major drivers of intestinal mucosal injury and epithelial structural disruption. The intestinal tract serves as the primary site for digestion and absorption. Villus height, crypt depth, and the villus height-to-crypt depth (V/C) ratio are critical indicators reflecting the digestive and absorptive ability of the small intestine. Higher villus height and lower crypt depth indicate improved nutrient utilization and accelerated maturation of intestinal epithelial tissue [37]. Moreover, a higher villus V/C ratio represents better structural integrity of the intestinal mucosa. In the present study, ICGA supplementation significantly increased villus height and the V/C ratio, while crypt depth showed no significant differences among treatments. Ma et al. reported that weaning stress induces intestinal oxidative damage and impairs small intestinal morphology and histology, characterized by villus shortening, villous fracture, and increased crypt depth, accompanied by downregulated mRNA expression of tight junction proteins [38]. Zhou et al. also demonstrated that ICGA supplementation improved villus height and the V/C ratio and growth performance in broilers [24]. These findings are consistent with our results, indicating that ICGA exerts pre-protective effects against LPS-challenged intestinal mucosal damage by stabilizing the structural integrity of the intestinal epithelium.

Oxidative stress-induced disturbance of intestinal morphological structure further impairs the function of the intestinal physical barrier, whose integrity is largely maintained by tight junction proteins [39]. Tight junction proteins, including Occludin and ZO-1, are indispensable components of the intestinal barrier, playing pivotal roles in preserving epithelial integrity, regulating ion transport, and controlling intestinal permeability [40]. Our results showed that LPS-induced oxidative stress markedly downregulated the gene expression of ZO-1, Occludin, and Claudin-1, leading to increased intestinal permeability and disrupted barrier homeostasis. In contrast, ICGA supplementation significantly upregulated the gene expression of these tight junction proteins. Combined with the morphological findings from intestinal tissue sections, ICGA could effectively inhibit oxidative stress-mediated intestinal barrier damage. By upregulating the expression of intestinal tight junction proteins, ICGA modulates intestinal water-salt metabolic balance, alleviates stress-induced intestinal exudation and diarrhoeal symptoms, and ultimately maintains the structural and functional integrity of the intestinal epithelium in lambs under LPS challenge.

### 4.4. ICGA Regulated the Intestinal Microbial Community Abundance of Lamb

The intestinal microbiota constitutes a complex microbial community inhabiting the intestinal tract, which plays important functions in maintaining intestinal barrier integrity and provides multiple benefits for the host, including pathogen defence, efficient nutrient utilization, and secretion of bioactive metabolites that inhibit competing harmful microbes [41]. Polyphenols have been widely demonstrated to modulate gut microbial composition and diversity in multiple models. In an in vitro colonic model, cocoa polyphenols improve gut microbiota diversity by elevating α-diversity indices, including Chao1 and Shannon [42]. Consistent with the polyphenol effects, our study showed that ICGA supplementation markedly reversed the LPS-challenged reductions in the α-diversity indices, including Chao1, Shannon, and PD-whole-tree. Polyphenols selectively suppress the proliferation of opportunistic pathogenic bacteria such as *Proteobacteria* by disrupting bacterial membrane structure and inhibiting metabolic enzymes, while beneficial taxa such as *Bacteroidetes* show greater tolerance to polyphenols [43]. Moreover, polyphenols serve as fermentable substrates for gut microbiota and are biotransformed into small phenolic metabolites, which further support the growth of beneficial microbes and stimulate short-chain fatty acid production [15,44]. Since intestinal inflammation reduces microbial diversity, the protective effect of polyphenols on the intestinal epithelium helps preserve a stable habitat for microbial colonization, thereby maintaining or restoring gut microbial diversity. In the present study, compared with the LPS group, ICGA supplementation significantly increased the relative abundance of *Bacteroidetes* and decreased the abundance of *Proteobacteria*. *Proteobacteria* are recognized as a signature phylum of gut dysbiosis, which includes many pathogenic bacteria closely associated with intestinal disorders [45].

Notably, the relative abundance of *Firmicutes* was higher in the LPS-challenged group than in the CON group and ICGA2 group. This observation differs from findings reported in chronic intestinal inflammation models. A plausible explanation is that our sampling was performed at the acute stage after LPS intraperitoneal challenge. During acute inflammatory stimulation, certain members of Firmicutes may undergo transient proliferation as a stress-responsive compensatory reaction in the ileum [46]. As a lactic-acid-producing commensal bacterium belonging to *Firmicutes*, *Sharpea azabuensis* may undergo transient compensatory proliferation in response to acute LPS stimulation. The *Firmicutes/Bacteroidetes* (F/B) ratio is widely used to evaluate the status of gut microbiota and host metabolic capacity. *Firmicutes* are mainly responsible for the degradation of dietary fibre and production of short-chain fatty acids [47], whereas *Bacteroidetes* preferentially decompose complex polysaccharides [48]. Changes in the F/B ratio reflect the shift of microbial metabolic function under stress. In the present study, the shift in F/B ratio in the LPS group represented a transient microbial adaptive response at the early acute inflammatory stage. ICGA supplementation further regulated this ratio, supporting the recovery of gut microbial balance.

Previous research reported that the relative abundance of *Proteobacteria* was significantly higher in diarrhoeal lambs than in healthy counterparts [49]. Jin et al. performed integrated metagenomic and transcriptomic analyses and revealed that the relative abundance of *Proteobacteria* was significantly positively correlated with the TLR4/MyD88/NF-κB inflammatory signaling pathway [50]. Meanwhile, the mRNA expression levels of downstream inflammatory cytokines increased. Wang et al. found that increased immunoglobulin concentrations were accompanied by enriched intestinal *Bacteroidetes* and *Bacteroides*, and Spearman correlation analysis showed significant positive correlations between *Bacteroidetes* abundance and CAT activity as well as T-AOC levels [51]. Representative strains within *Bacteroidetes*, such as *Bacteroides* ovatus, promote IgA production via a T cell-dependent pathway [52]. With *Actinobacteria*, Bifidobacterium bifidum can bind to TLR4 and drive the differentiation of B cells into IgA-secreting plasma cells, thereby enhancing immune function [53]. Collectively, these results indicated that Bacteroidetes and Actinobacteria could regulate the antioxidant capacity and immune competence of the host. This study showed that Spearman correlation analysis revealed that *Bacteroidetes* and *Aeriscardovia* were positively correlated with immunoglobulin concentrations and antioxidant enzyme activities, suggesting that these two phyla may participate in host antioxidant and immune regulation, whereas *Proteobacteria* exhibited a positive correlation with the levels of inflammatory cytokines.

### 4.5. Limitations

The present study has several limitations. First, an ICGA-alone group without LPS challenge was not included in the experimental design, making it difficult to separate the direct effects of ICGA supplementation from its protective effects against LPS-challenged intestinal injury. Second, all biological samples were collected at a single time point following LPS challenge; therefore, we cannot characterize the dynamic profiles of intestinal damage, inflammatory response, and signaling pathway activation throughout the acute stress process. Third, the conclusions regarding the TLR4/MyD88/NF-κB and Nrf2/HO-1 pathways are only supported by mRNA expression data. Further protein quantification and phosphorylation analysis are required to validate the regulatory changes at the translational and post-translational levels. Fourth, the sample size for 16S rRNA gene sequencing was relatively small (*n* = 4 per group), which may reduce statistical power and limit the extrapolation of the observed microbiota results to broader lamb populations. Fifth, the basal ICGA concentration in the basal diet was not measured in the present study. We acknowledge that future investigations should quantify the actual ICGA concentration in experimental diets. Sixth, the ICGA dose was chosen with reference to an in vitro ruminal fermentation study and further validated by our preliminary experiments. We recognize the limitation that these doses have not been optimized by in vivo dose–response assays.

## 5. Conclusions

In conclusion, this study demonstrates that ICGA modulates intestinal microbiota composition in LPS-acute inflammatory challenged lambs and increases the relative abundance of intestinal microbes. Meanwhile, ICGA can inhibit the expression of genes related to the TLR4/MyD88/NF-κB signaling pathway and reduce the mRNA levels of TNF-α, IL-1β, and IL-6, ultimately improving intestinal tight junction proteins *Occludin* and *ZO-1* expression. Additionally, ICGA partially mitigates LPS-induced acute inflammatory challenge-induced oxidative injury associated with altered Nrf2/HO-1 signaling pathway and enhances systemic antioxidant capacity. Collectively, because both ICGA doses had similar effects, these results indicate that the supplemental level of ICGA was 1.5 g/kg–2.0 g/kg diet for weaning lambs.

## Figures and Tables

**Table 1:** Composition and nutrient levels of basal diets (DM basis).

Peanut vine	20.00
Corn grain	40.00
Soybean meal	16.00
Cottonseed meal	19.40
premix	4.00
NaCl	0.20
CaHPO_4_	0.40
Total	100
Nutrient levels	
ME, MJ/kg	10.59
CP, %	18.33
EE, %	3.24
NDF, %	28.15
ADF, %	12.51
Ca, %	0.58
P, %	0.30

Notes: The premix provided per kg of dry matter: VA 9000 IU, VD3 800 IU, VE 27 IU, VB1 4 mg, VB2 3.6 mg, VB5 40 mg, VB12 0.02 mg, Cu 10 mg, Fe 350 mg, Zn 45 mg, Mn 45 mg, Ca 0.8 g, P 0.08 g, I 0.4 mg, Se 0.4 mg, Co 0.2 mg. ME = Metabolizable energy was calculated based on the total digestible nutrients of the feed ingredient (ME = 0.82 × 4.409 × TDN) and their proportions, referring to NRC (2007) [21]; CP = crude protein. EE = ether extract. NDF = Neutral detergent fiber. ADF = Acid-detergent fiber. The ME is a calculated value, while other indicators are measured values.

**Table 2:** Primer sequences.

Genes	Accession Number	Primer Sequence (5′–3′)	Product Length/bp
*GAPDH*	NM_001190390.1	F: TGGCATCGTGGAGGGACTTA R: CATCATACTTGGCAGGTTTCTCC	266
*IL-1β*	NM_001009465.2	F: CAAAGTCTACCCCAAGAGGAATATG R: GAAGTGCTGATGTACCAGTTAGGG	114
*IL-6*	NM_001314054.1	F: GGGAAATCGTGGAAATGAGAAA R: CAGTTTGGTAGCATCCATCATTTCT	129
*TNF-α*	NM_001024860.1	F: CGTTGTAGCCAACATCAGCG R: TGGTAGGAGACTGCAATGCG	219
*TLR4*	NM_001135930.1	F: GGGCTTAGAGCAGCTAGAACACC R: TAAAGATGCCGTGGAAGACAAT	146
*MyD88*	NM_001166183.1	F: TACCTGCAAAGCAAGGAATGTG R: GGAACTCTTTCTTCATTGGCTTGT	115
*NF-kBp65*	XM_027959295.2	F: CTGCCGGGTAAATCGGAACT R: GCCTGGTCCCGTGAAATACA	109
*ZO-1*	XM_042235170.1	F: CCTGCTGGGACTAATAGCCA R: CAGCCATTCGCATCTTCTGT	170
*Occludin*	XM_015101255.3	F: GCCTGTGTTGCCTCCACTCTTG R: CCGTAGCCATAACCATAGCCATAGC	143
*Claudin-1*	NM_001185016.1	F: TTCCACCGTAATGACCCTCCAGAG R: AAGGCACATCATTCCACCACAAGAG	156
*Keap1*	XM_027969637.3	F: CCATCGGCATTGCCAACTTC R: AAGACCTCAGACTCGCAACG	187
*HO-1*	XM_027967703.3	F: ACAGGCCACCAAGTGCTATG R: TCAGGGCCTTCTGAGCAATC	143
*Nrf2*	XM_060417984.1	F: CTTTACACTGGGCCACGGAA R: GGGCTCTCTGGGTTTGTGTT	193

Notes: F: forward primer; R: reverse primer. All gene accession numbers listed above are from *Ovis aries*.

## Data Availability

The raw 16S rRNA sequencing data generated in this study have been deposited into the NCBI Sequence Read Archive (SRA) (accession number: PRJNA1534686). All other datasets generated and analyzed in this study are available from the corresponding author (gyx310@163.com) upon reasonable request.

## References

[1] Li C., Xu Y., Jia J., Weng X., Zhang Y., Peng J., An X., Wang G. (2025). Impacts of Early Weaning on Lamb Gut Health and Immune Function: Short-Term and Long-Term Effects. Animals.

[2] Orgeur P., Bernard S., Naciri M., Nowak R., Schaal B., Lévy F. (1999). Psychobiological Consequences of Two Different Weaning Methods in Sheep. Reprod. Nutr. Dev..

[3] Zhao F., He W., Guo D., Wang W., Zhang A. (2024). Effects of quercetin on serum and intestinal antioxidant indices and intestinal barrier function in weaned lambs. J. Heilongjiang Bayi Agric. Univ..

[4] Liu L., Chen D., Yu B., Luo Y., Huang Z., Zheng P., Mao X., Yu J., Luo J., Yan H. (2021). Influences of Selenium-Enriched Yeast on Growth Performance, Immune Function, and Antioxidant Capacity in Weaned Pigs Exposure to Oxidative Stress. BioMed Res. Int..

[5] Ferronato G., Cattaneo L., Trevisi E., Liotta L., Minuti A., Arfuso F., Lopreiato V. (2022). Effects of Weaning Age on Plasma Biomarkers and Growth Performance in Simmental Calves. Animals.

[6] Li C., Wang W., Liu T., Zhang Q., Wang G., Li F., Li F., Yue X., Li T. (2018). Effect of Early Weaning on the Intestinal Microbiota and Expression of Genes Related to Barrier Function in Lambs. Front. Microbiol..

[7] Weary D.M., Jasper J., Hötzel M.J. (2008). Understanding Weaning Distress. Appl. Anim. Behav. Sci..

[8] Kaur R., Kaur J., Mahajan J., Kumar R., Arora S. (2014). Oxidative Stress--Implications, Source and Its Prevention. Environ. Sci. Pollut. Res. Int..

[9] McGarry T., Biniecka M., Veale D.J., Fearon U. (2018). Hypoxia, Oxidative Stress and Inflammation. Free Radic. Biol. Med..

[10] Nathan C., Ding A. (2010). Nonresolving Inflammation. Cell.

[11] Rudrapal M., Rakshit G., Singh R.P., Garse S., Khan J., Chakraborty S. (2024). Dietary Polyphenols: Review on Chemistry/Sources, Bioavailability/Metabolism, Antioxidant Effects, and Their Role in Disease Management. Antioxidants.

[12] Mohammadi A., Blesso C.N., Barreto G.E., Banach M., Majeed M., Sahebkar A. (2019). Macrophage Plasticity, Polarization and Function in Response to Curcumin, a Diet-Derived Polyphenol, as an Immunomodulatory Agent. J. Nutr. Biochem..

[13] Myint K.Z., Zhou Z., Shi Q., Chen J., Dong X., Xia Y. (2023). Stevia Polyphenols, Their Antimicrobial and Anti-Inflammatory Properties, and Inhibitory Effect on Digestive Enzymes. Molecules.

[14] Scarpati M.L., Guiso M. (1964). Structure of the Three Dicaffeoyl-Quinic Acids of Coffee (Isochlorogenic Acid). Tetrahedron Lett..

[15] Yuan Y., Wu L., Hao Q., Guo Y. (2025). Research progress on absorption and metabolism of isochlorogenic acid and its application in livestock and poultry production. Chin. J. Anim. Nutr..

[16] Wang Q., Xiao L. (2019). Isochlorogenic Acid a Attenuates Acute Lung Injury Induced by LPS via Nf-κB/NLRP3 Signaling Pathway. Am. J. Transl. Res..

[17] Lai X., Fan P., Deng H., Jia G., Zuo Z., Hu Y., Wang Y., Cai D., Gou L., Wen Y. (2024). Effects of Isochlorogenic Acid a on Mitochondrial Dynamics Imbalance and RLR Damage in PAM Cells Induced by Combined Mycotoxins. Toxicology.

[18] Cheng W., Hao Q., Yuan Y., Zhao P., Qian Z., Guo Y., Tian Y. (2025). Protective Effect of Isochlorogenic Acid Ex-tracted from Stevia rebaudiana against Hydrogen Peroxide-Induced Intestinal Epithelial Cell Injury. Food Sci..

[19] Li S., Jiao T., Qi S., Zhang X., Wang H., Zhao S. (2024). In vitro study on effects of isochlorogenic acid supplemen-ta-tion in sheep diets on nutrient digestion and rumen fermentation. Acta Agric. Zhejiangenis.

[20] Wang K., Chen X., Zuo L., Pan C., Liu G., Zhang X., Du J., Zhang C., Zhang B., Wang Z. (2021). Dietary Sodium Butyrate Protects Lipopolysaccharide-Induced Inflammatory Response in Lambs through Inhibiting TLR4/NF-κB Signalling Pathway. Ital. J. Anim. Sci..

[21] National Research Council (NRC) Committee on Nutrient Requirements of Small Ruminants (2007). Nutrient Requirements of Small Ruminants: Sheep, Goats, Cervids, and New World Camelids.

[22] Guo Y., Yang R., Duan C., Wang Y., Hao Q., Ji S., Yan H., Zhang Y., Liu Y. (2023). Effect of Dioscorea Opposite Waste on Growth Performance, Blood Parameters, Rumen Fermentation and Rumen Bacterial Community in Weaned Lambs. J. Integr. Agric..

[23] Zhao L., Pan F., Lin W., Xu M., Bao X., Chen Y., Wang C., Lian Y. (2021). Main components of stevia residue ex-tract and their antioxidant activities. Food Sci..

[24] Zhou S., Jiang H., Wang Y., Yan A., Liu G., Liu S., Chen B. (2025). Dietary Supplementation with Isochlorogenic Acid Improves Growth Performance and Intestinal Health of Broilers. Anim. Nutr..

[25] Jiang H., Zhou S., Wang Y., Zhao Z., Wei X., Yan A., Liu G., Liu S., Chen B. (2024). Effects of isochorogenic acid on growth performance, nutrient apparent metabolic rates of broilers and antioxidant capacity of broiler under lipopolysaccharide stress. Chin. J. Anim. Nutr..

[26] Salman S., Khol-Parisini A., Schafft H., Lahrssen-Wiederholt M., Hulan H.W., Dinse D., Zentek J. (2009). The Role of Dietary Selenium in Bovine Mammary Gland Health and Immune Function. Anim. Health Res. Rev..

[27] Yin X., Ji S., Duan C., Ju S., Zhang Y., Yan H., Liu Y. (2021). Rumen Fluid Transplantation Affects Growth Performance of Weaned Lambs by Altering Gastrointestinal Microbiota, Immune Function and Feed Digestibility. Animal.

[28] Weißmüller S., Schmidt C., Heinz C.C. (2026). Modes of (Inter)Actions of Polyvalent Immunoglobulins: Nonclinical and Clinical Research in Severe Bacterial Infections. Biomedicines.

[29] Huang X., Shen H., Li Z., Fu T., Wei X., Qiu S. (2025). Isochlorogenic Acid a Attenuated Periodontitis by Inhibiting Inflammation via ACE/PGK1/STAT1 Signaling. J. Agric. Food Chem..

[30] Shi L., Dai Y., Jia B., Han Y., Guo Y., Xie T., Liu J., Tan X., Ding P., Li J. (2019). The Inhibitory Effects of Qingchang Wenzhong Granule on the Interactive Network of Inflammation, Oxidative Stress, and Apoptosis in Rats with Dextran Sulfate Sodium-Induced Colitis. J. Cell. Biochem..

[31] Li T., Zhang T., Gao H., Liu R., Gu M., Yang Y., Cui T., Lu Z., Yin C. (2021). Tempol Ameliorates Polycystic Ovary Syndrome through Attenuating Intestinal Oxidative Stress and Modulating of Gut Microbiota Composition-Serum Metabolites Interaction. Redox Biol..

[32] Wang X., Li Y., Yin L., Fan S., Zhang S., Ou H., Wang J., Li H. (2026). The effect of abdominal Tuina on intestinal barrier function in IBS-D rats through the regulation of the TLR4/MyD88/NF-κB signaling pathway. Liaoning J. Tradit. Chin. Med..

[33] Wang H.-N., Shen Z., Liu Q., Hou X.-Y., Cao Y., Liu D.-H., Jiang H., Du H.-Z. (2020). Isochlorogenic Acid (ICGA): Natural Medicine with Potentials in Pharmaceutical Developments. Chin. J. Nat. Med..

[34] Qu S., Tian Q., Mei H., Li Z., Li Y., Ma X., Gao F., Yu M. (2022). Effects of Magnolo on growth performance, liver antioxidant function and lipid metabolism of weaned piglets. Chin. J. Anim. Nutr..

[35] Dinkova-Kostova A.T., Kostov R.V., Canning P. (2017). Keap1, the Cysteine-Based Mammalian Intracellular Sensor for Electrophiles and Oxidants. Arch. Biochem. Biophys..

[36] Chen A., Ma T., Zhong Y., Deng S., Zhu S., Fu Z., Huang Y., Fu J. (2024). Effect of Tea Polyphenols Supplement on Growth Performance, Antioxidation, and Gut Microbiota in Squabs. Front. Microbiol..

[37] Zuo L., Chen X., Wang K., Jiang N., Zhang A. (2020). Effects of Sodium Butyrate on Gastrointestinal Tract Development of Weaned Lambs. J. Anim. Nutr..

[38] Ma J., Fan X., Zhang W., Zhou G., Yin F., Zhao Z., Gan S. (2023). Grape Seed Extract as a Feed Additive Improves the Growth Performance, Ruminal Fermentation and Immunity of Weaned Beef Calves. Animals.

[39] Li B., Zhang Y., Liu X., Zhang Z., Zhuang S., Zhong X., Chen W., Hong Y., Mo P., Lin S. (2024). Traditional Chinese Medicine Pien-Tze-Huang Ameliorates LPS-Induced Sepsis through Bile Acid-Mediated Activation of TGR5-STAT3-A20 Signalling. J. Pharm. Anal..

[40] Furuse M., Itoh M., Hirase T., Nagafuchi A., Yonemura S., Tsukita S., Tsukita S. (1994). Direct Association of Occludin with ZO-1 and Its Possible Involvement in the Localization of Occludin at Tight Junctions. J. Cell Biol..

[41] Yoon M.Y., Lee K., Yoon S.S. (2014). Protective Role of Gut Commensal Microbes against Intestinal Infections. J. Microbiol..

[42] Nagata R., Sato S., Kilua A., Fukuma N., Nakayama Y., Kitazono E., Aoyama T., Han K.-H., Fukushima M. (2021). Combined Effects of BARLEYmax and Cocoa Polyphenols on Colonic Microbiota and Bacterial Metabolites in Vitro. Food Sci. Biotechnol..

[43] Wu L., Gao W., Zhao P., Shi Z., Zhao J., Xu M., Zhu Z., Song W. (2023). Effects of stevia isochlorogenic salt on production performances, immune and serum hormones indexes of laying hens in late laying period. Heilongjiang Anim. Sci. Vet. Med..

[44] Jiang H., Zhou S., Wang Y., Gao L., Zhao M., Liu G., Liu S., Chen B. (2025). Dietary Isochlorogenic Acid Ameliorates Intestinal Inflammation and Barrier Damage by Modulating Gut Microbiota and Promoting L-Lysine Metabolism in LPS-Challenged Broilers. Anim. Nutr..

[45] Shin N.-R., Whon T.W., Bae J.-W. (2015). *Proteobacteria*: Microbial Signature of Dysbiosis in Gut Microbiota. Trends Biotechnol..

[46] Shen J., Wang S., Xia H., Han S., Wang Q., Wu Z., Zhuge A., Li S., Chen H., Lv L. (2023). Akkermansia Muciniphila Attenuated Lipopolysaccharide-Induced Acute Lung Injury by Modulating the Gut Microbiota and SCFAs in Mice. Food Funct..

[47] Jiang H., Dong J., Jiang S., Liang Q., Zhang Y., Liu Z., Ma C., Wang J., Kang W. (2020). Effect of *Durio zibethinus* Rind Polysaccharide on Functional Constipation and Intestinal Microbiota in Rats. Food Res. Int..

[48] Thomas F., Hehemann J.-H., Rebuffet E., Czjzek M., Michel G. (2011). Environmental and Gut Bacteroidetes: The Food Connection. Front. Microbiol..

[49] Zhong T., Wang Y., Wang X., Freitas-de-Melo A., Li H., Zhan S., Wang L., Cao J., Dai D., Guo J. (2022). Diarrhea in Suckling Lambs Is Associated with Changes in Gut Microbiota, Serum Immunological and Biochemical Parameters in an Intensive Production System. Front. Microbiol..

[50] Jin C., Wu S., Liang Z., Zhang J., Lei X., Bai H., Liang G., Su X., Chen X., Wang P. (2024). Multi-Omics Reveal Mechanisms of High Enteral Starch Diet Mediated Colonic Dysbiosis via Microbiome-Host Interactions in Young Ruminant. Microbiome.

[51] Wang K., Zhu Q., Kong X., Song M., Azad M.A.K., Xiong L., Zheng Y., He Q. (2020). Dietary Probiotics or Synbiotics Supplementation during Gestation, Lactation, and Nursery Periods Modifies Colonic Microbiota, Antioxidant Capacity, and Immune Function in Weaned Piglets. Front. Vet. Sci..

[52] Yang C., Mogno I., Contijoch E.J., Borgerding J.N., Aggarwala V., Li Z., Siu S., Grasset E.K., Helmus D.S., Dubinsky M.C. (2020). Fecal IgA Levels Are Determined by Strain-Level Differences in *Bacteroides ovatus* and Are Modifiable by Gut Microbiota Manipulation. Cell Host Microbe.

[53] Zhao M., Liang X., Meng Y., Lu H., Lin K., Gong P., Liu T., Yi H., Pan J., Zhang Y. (2024). Probiotics Induce Intestinal IgA Secretion in Weanling Mice Potentially through Promoting Intestinal APRIL Expression and Modulating the Gut Microbiota Composition. Food Funct..

